# Nitrogen Photoelectrochemical Reduction on TiB_2_ Surface Plasmon Coupling Allows Us to Reach Enhanced Efficiency of Ammonia Production

**DOI:** 10.1021/acscatal.3c03210

**Published:** 2023-08-03

**Authors:** Anna Zabelina, Elena Miliutina, Jakub Dedek, Andrii Trelin, Denis Zabelin, Rashid Valiev, Ruslan Ramazanov, Vasilii Burtsev, Daniela Popelkova, Martin Stastny, Vaclav Svorcik, Oleksiy Lyutakov

**Affiliations:** † Department of Solid State Engineering, University of Chemistry and Technology, 16628 Prague, Czech Republic; ‡ Department of Chemistry, University of Helsinki, FI-00014 Helsinki, Finland; § Institute of Inorganic Chemistry, Czech Academy of Sciences, 250 68 Husinec-Rez, Czech Republic

**Keywords:** photoelectrochemical nitrogen reduction, plasmon coupling, TiB_2_, NH_3_ production, hybrid photocatalyst

## Abstract

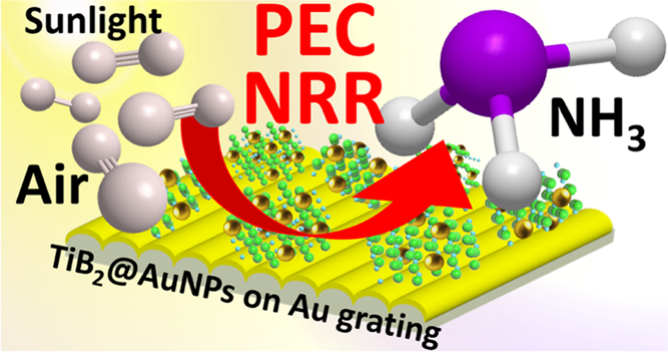

Ammonia is one of the most widely produced chemicals worldwide, which is consumed in the fertilizer industry and is also considered an interesting alternative in energy storage. However, common ammonia production is energy-demanding and leads to high CO_2_ emissions. Thus, the development of alternative ammonia production methods based on available raw materials (air, for example) and renewable energy sources is highly demanding. In this work, we demonstrated the utilization of TiB_2_ nanostructures sandwiched between coupled plasmonic nanostructures (gold nanoparticles and gold grating) for photoelectrochemical (PEC) nitrogen reduction and selective ammonia production. The utilization of the coupled plasmon structure allows us to reach efficient sunlight capture with a subdiffraction concentration of light energy in the space, where the catalytically active TiB_2_ flakes were placed. As a result, PEC experiments performed at −0.2 V (vs. RHE) and simulated sunlight illumination give the 535.2 and 491.3 μg h^–1^ mg_cat_^–1^ ammonia yields, respectively, with the utilization of pure nitrogen and air as a nitrogen source. In addition, a number of control experiments confirm the key role of plasmon coupling in increasing the ammonia yield, the selectivity of ammonia production, and the durability of the proposed system. Finally, we have performed a series of numerical and quantum mechanical calculations to evaluate the plasmonic contribution to the activation of nitrogen on the TiB_2_ surface, indicating an increase in the catalytic activity under the plasmon-generated electric field.

## 1 Introduction

Ammonia (NH_3_) is an important industrial chemical that plays a vital role in agriculture and pharmacy and was recently considered a carbon-free solar energy storage material.^1−3^ It should be noted that the common ammonia production is based on the energy- and material-demanding Haber–Bosch processes and actually consumes 1–3% of the world’s electrical energy and 5% of the world’s natural gas production.^4,5^ Thus, the development of an alternative, less energy, and irreplaceable raw material-consuming approach is urgently needed for large-scale ammonia production and utilization.^6−9^ One of the more promising ways is the electrochemical reduction of N_2_, which can be performed under mild conditions (that is, at room temperature, RT, and under atmospheric pressure).^10−12^ However, due to the inertness of the strong N≡N triple bonds, the efficiency of the electrochemical N_2_ reduction is still far from a commercially acceptable level.^13^ To overcome this barrier, various catalytically active materials were proposed and tested, including noble and common metal-based materials, binary or ternary compounds, and non-metal-based materials.^14−19^ However, despite intensive research and progress in this field, currently reported values of process efficiency and related NH_3_ production rate still require further improvements.

To reduce the energy demands of the electrochemical reduction of N_2_ and partially compensate for the insufficient faradaic efficiency, the utilization of a photoelectrochemical approach (PEC) approach was proposed.^20−22^ PEC processes allow the combination of the merits of electrocatalysis and photocatalysis and represent an attractive prospect for the production of ammonia from pure or atmospheric nitrogen under mild conditions.^23−25^ Recently, the meticulous combination of materials responsible for light absorption and nitrogen fixation/activation allows us to reach the faradaic efficiency of nitrogen reduction reaction (NRR) even above 50%.^26,27^ In this regard, especially interesting and effective is the coupling of “common” redox-active materials with plasmon-active nanostructures, which serve for light energy absorption and subdiffraction focusing.^28^ For example, materials such as Nb-SrTiO_3_, BSi/Cr, TiO_2_, and p-Si were loaded with plasmon-active nanoparticles and successfully utilized for NRR.^29,30^ These approaches allow us to reach the highest reported values of the NH_3_ synthesis rate with the utilization of only renewable sunlight energy in the nitrogen technical cycle.^31^

In the development of hybrid materials for plasmon-assisted NRR (i.e., materials constructed from redox and plasmon-active parts), the main attention is focused on the “redox” part, while the design of plasmonic (nano)structures often goes unnoticed. On the other hand, the utilization of several simple approaches, well known from alternative plasmonic applications, can significantly (up to several orders of magnitude)^24,32^ increase the local value of plasmon-related energy, potentially enhancing the NRR efficiency of the carefully designed hybrid catalyst. As an example, the coupling of two nanostructures, supporting the excitation of local (LSP, localized surface plasmon) and traveled (SPP, surface plasmon polariton) plasmons, can be mentioned as the way to significant local energy increase.

In this work, we propose the utilization of LSP–SPP coupling for the triggering of NRR catalytic activity of thin MBene nanostructures (TiB_2_), which were previously demonstrated to be catalytically active in NRR.^33^

## 2 Experimental Section

### 2.1 Materials and Sample Preparation

A detailed description of the used materials, sample preparation, and characterization is given in the Supporting Information (SI) part. Briefly, layered TiB_2_ sheets were prepared from the corresponding powder using a high-intensity cavitation field in an ultrasonic reactor. The gold nanoparticles (AuNPs), able to support the LSP excitation, were subsequently synthesized on the TiB_2_ surface by the ultrasound-assisted reduction of the HAuCl_4_ salt dissolved in deionized water. The created TiB_2_@AuNP hybrid materials were subsequently deposited on the surface of the gold grating, able to support the SPP excitation.

### 2.2 Electrochemical and Photoelectrochemical and Solely Photochemical NRR

Electrochemical measurements were carried out using a Palm Sens 4 potentiostat (Palm Instruments, The Netherlands) controlled by the PSTrace 5.9 program using a three-electrode two-compartment electrolytic cell (H-type), which was separated by a Nafion 117 membrane. The Au grating and Au grating/TiB_2_@AuNP samples were used as a working electrode, and the Ag/AgCl electrode (BVT Technologies, CZ) was used as a reference electrode. As a counter electrode, a platinum wire electrode (BASi) with a 0.7 × 0.6 cm^2^ active surface area was used. All photoelectrocatalytic N_2_ reduction experiments were carried out in a 0.1 M Na_2_SO_4_ solution with the addition of an ionic liquid (20 wt %) ([C4mpyr][eFAP]) to block HER. The electrolyte was purged with different gases (N_2_, Ar, air, and a mixture of Ar/N_2_) for 30 min before the experiment and then continuously purged with nitrogen during the experiments. Additionally, electrochemical measurements were performed without and with the illumination of the sample’s surface using the solar simulator (Solar Simulator SciSun-300, Class AAA, and the intensity on the sample surface was adjusted to 100 mW cm^–2^). The solely photocatalytic nitrogen reduction was also performed in a 0.1 M Na_2_SO_4_ mixture solution containing 20 wt % ([C4mpyr][eFAP]) with the utilization of a SciSun-300 solar simulator.

The amount of ammonia produced was quantitatively determined by the ammonia photometric kit test (0.010–3.00 mg L^–1^ (NH_4_^+^), Spectroquant, Supelco, Merck) by the procedure suggested by the supplier. The NRR faradaic efficiency was subsequently calculated using the following equation: , where *F* is the Faraday constant, *C*_NH_3__ is the total amount of NH_3_ (measured by the photometric test), *V* is the electrolyte volume, and *Q* is the quantity of the applied electricity.

As a control, isotope labeling experiments were performed with the utilization of ^15^N_2_ instead of ^14^N_2_ for electrolyte purging (see the SI for details). The results were analyzed using ^1^H NMR measurements with aqueous solutions of NH_4_Cl and (^15^NH_4_)_2_SO_4_ as standards. Furthermore, the potential yield of N_2_H_4_ was determined using the Watt and Chrisp method. Another set of control experiments (estimation of the impact of plasmon coupling on the NRR efficiency) includes the utilization of pristine TiB_2_ flakes and TiB_2_@AuNP structures deposited on the glass electrode surface, as well as pristine TiB_2_ flakes deposited on the Au grating surface (instead of Au grating/TiB_2_@AuNP photoelectrode).

### 2.3 Calculation of the TiB_2_@AuNP Electronic Structure, Plasmon Energy Distribution, and NRR Pathway

The calculation of the valence and conductive band-gap positions of TiB_2_@AuNPs was performed using the combination of Tauc and Mott–Schottky plots (details are given in the SI). The distribution of plasmon-related electric field and related volumetric energy density increase in LSP–SPP-coupled hot spots were determined using finite-difference time-domain (FDTD) simulation using MEEP software. Subsequently, the free-energy profile in the plasmonic hot spot was estimated (details are given in the SI).

The obtained results were subsequently used in the density functional calculation of plasmon-assisted NRR pathways, performed in CP2K software.^34^ We considered the free-energy profile of the adsorption and subsequent hydrogenation of nitrogen molecules accompanied by releasing two ammonia molecules. Comparative DFT calculations were done under the applied external potential (or zero potential) and under a plasmon-generated uniform electric field (details are given in the SI).

## 3 Results and Discussion

### 3.1 Catalyst Preparation and Characterizations

**Figure 1:**
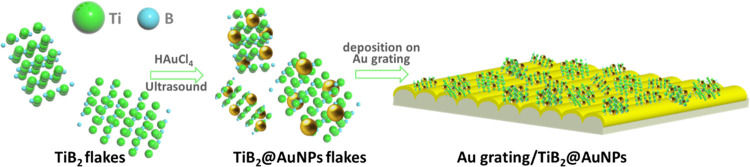
Schematic concept of the Au grating/TiB_2_@AuNP preparation: preparation of LSP excitation-supported AuNPs on the surface of redox-active TiB_2_ flakes and subsequent deposition on Au grating able to support SPP excitation. The resulting structure will ensure the LSP–SPP coupling in place of TiB_2_ flakes.

Our main experimental concept of sample creation with the TiB_2_ flake introduction into the LSP–SPP plasmonic hot spot(s) is schematically presented in Figure 1. We used the thin TiB_2_ nanostructure, with a quasi-two-dimensional (2D) geometry created by high-power ultrasonication. The thicknesses of the flakes were in the 3–4 nm range, providing a suitable spacer for efficient plasmonic coupling. The gold nanoparticles (AuNPs) were deposited on the TiB_2_ surface using the gold reduction performed directly in the TiB_2_ suspension. Characterization and control experiments of the created materials (Figures S1–S3 and related discussion) indicate that all AuNPs are deposited on the TiB_2_ surface, and there are no free-standing AuNPs in the solution. After carefully washing the TiB_2_@AuNP materials, the flakes were deposited on the surface of the plasmon-active grating using the previously optimized route.^32^ Due to the regularly patterned surface (Figure S4), the gold grating surface can support the excitation of SPP, which manifests itself as an absorption band located near 700 nm (Figure S5). In turn, the immobilized AuNPs are responsible for the excitation of LSP, located near 550 nm (Figure S3B). The spatial proximity of the Au grating and AuNP, ensured by the TiB_2_ “spacer”, can lead to a high local concentration of light energy, which supposedly could be responsible for the sufficient triggering of the TiB_2_ catalytic activity in NRR.

**Figure 2:**
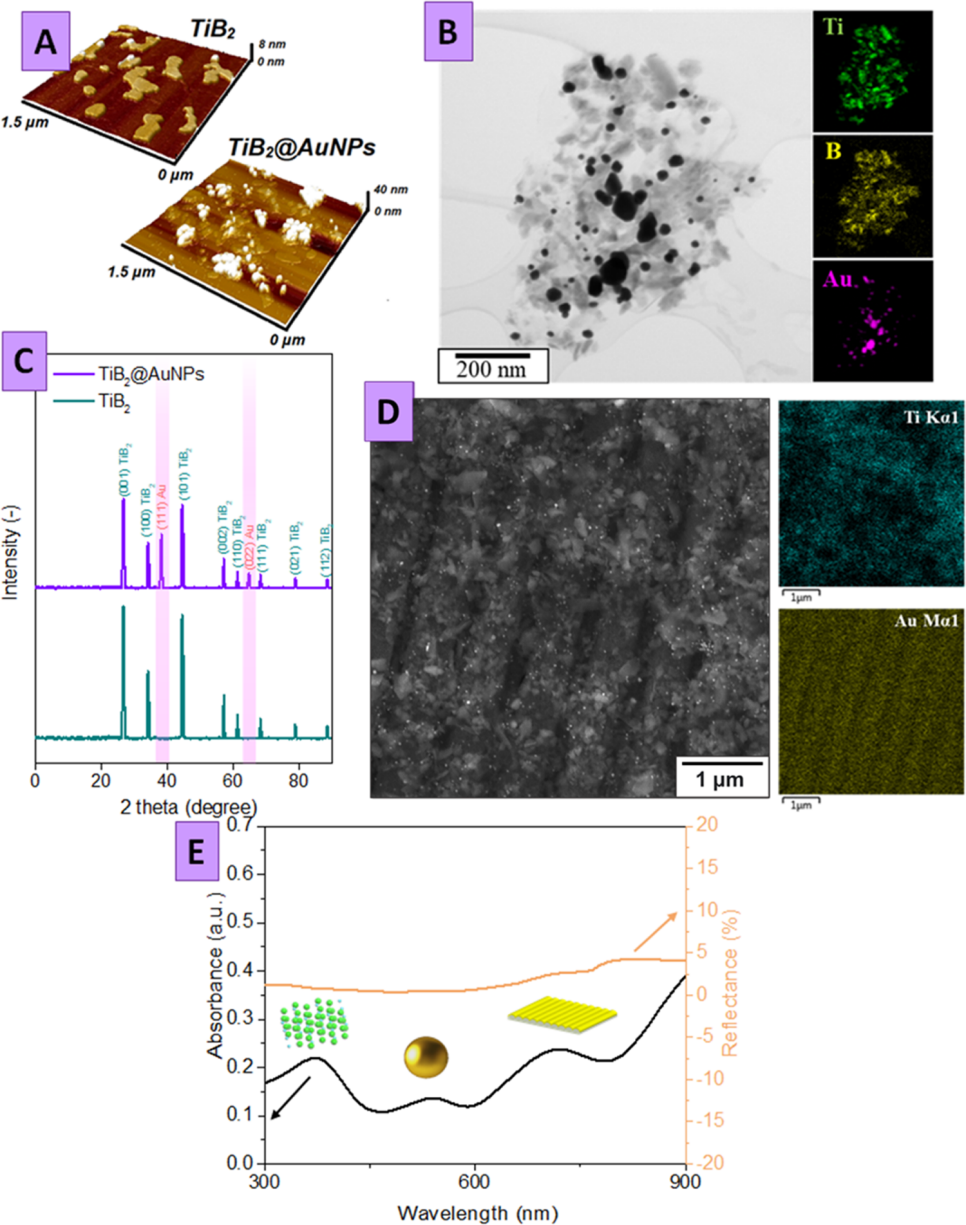
(A) AFM studied the morphology of TiB_2_ and TiB_2_@AuNP flakes deposited on the Si substrate; (B) TEM image of the TiB_2_@AuNP flake and corresponding EDX mapping of Ti, B, and Au; (C) XRD patterns of TiB_2_ and TiB_2_@AuNP flakes; (D) SEM–EDX measured spatial distribution of TiB_2_@AuNP flakes on the Au grating surface; and (E) UV–vis and reflection spectroscopy of Au grating/TiB_2_@AuNPs.

The surface morphology and high-resolution transmission electron microscope with the energy-dispersive X-ray (HRTEM–EDX)-measured distribution of AuNPs on the TiB_2_ flake surface are presented in Figure 2A,B. In particular, atomic force microscopy (AFM) scans reveal the appearance of characteristic morphological features on the TiB_2_ surface (Figure 2A), which, according to the EDX mapping, should be attributed to AuNPs. In turn, the separately performed X-ray diffraction (XRD) mapping reveals the multicrystalline structure of TiB_2_ (Figure 2C), with the presence of significant characteristic (001) phase reflexes [JCPDS No. 08-0121]. Even the presence of this phase has been predicted to be responsible for the sufficient NRR proceeding on the surface of TiB_2_ flakes.^33^ The fact that this phase was maintained even after the flake coupling with AuNPs was confirmed by XRD and HRTEM analyses.

The typical surface morphology of Au grating/TiB_2_@AuNP samples is presented in Figure 2D (and Figure S4), indicating the conservation of regular geometry and homogeneous distribution of TiB_2_@AuNP nanostructures on the Au grating surface. Both were well visible from the EDX mapping of the Ti distribution and additional Raman mapping presented in Figure S6. UV–vis absorption and reflection spectra of Au grating/TiB_2_@AuNP samples are shown in Figure 2E, which can be compared in Figure S7, showing the light reflection from the pristine Au grating. Several absorption bands, attributed to the intrinsic TiB_2_ light absorption (below 400 nm wavelength), excitation of LSP on the AuNP surface (near a 550 nm wavelength), and SPP on the gold grating surface (near a 700 nm wavelength) well overlapping with the sunlight spectrum, are visible from the UV–vis absorption spectra (Figure 2E). In addition, the results of reflection spectroscopy (Figure S7 vs. Figure 2E) reveal a significant decrease of the reflected light intensity after the electrophoretic deposition of TiB_2_@AuNPs on the Au grating surface. The decrease is due to the strengthening of light absorption through the LSP–SPP coupling and surface roughening, which ensure the multiple-photon surface scattering and related increase of their absorption probability. An extended range of the “absorbed” wavelengths and the simultaneous decrease in the reflected light intensity provide more efficient utilization of sunlight energy.

### 3.2 Photoelectrochemical Experiments

**Figure 3:**
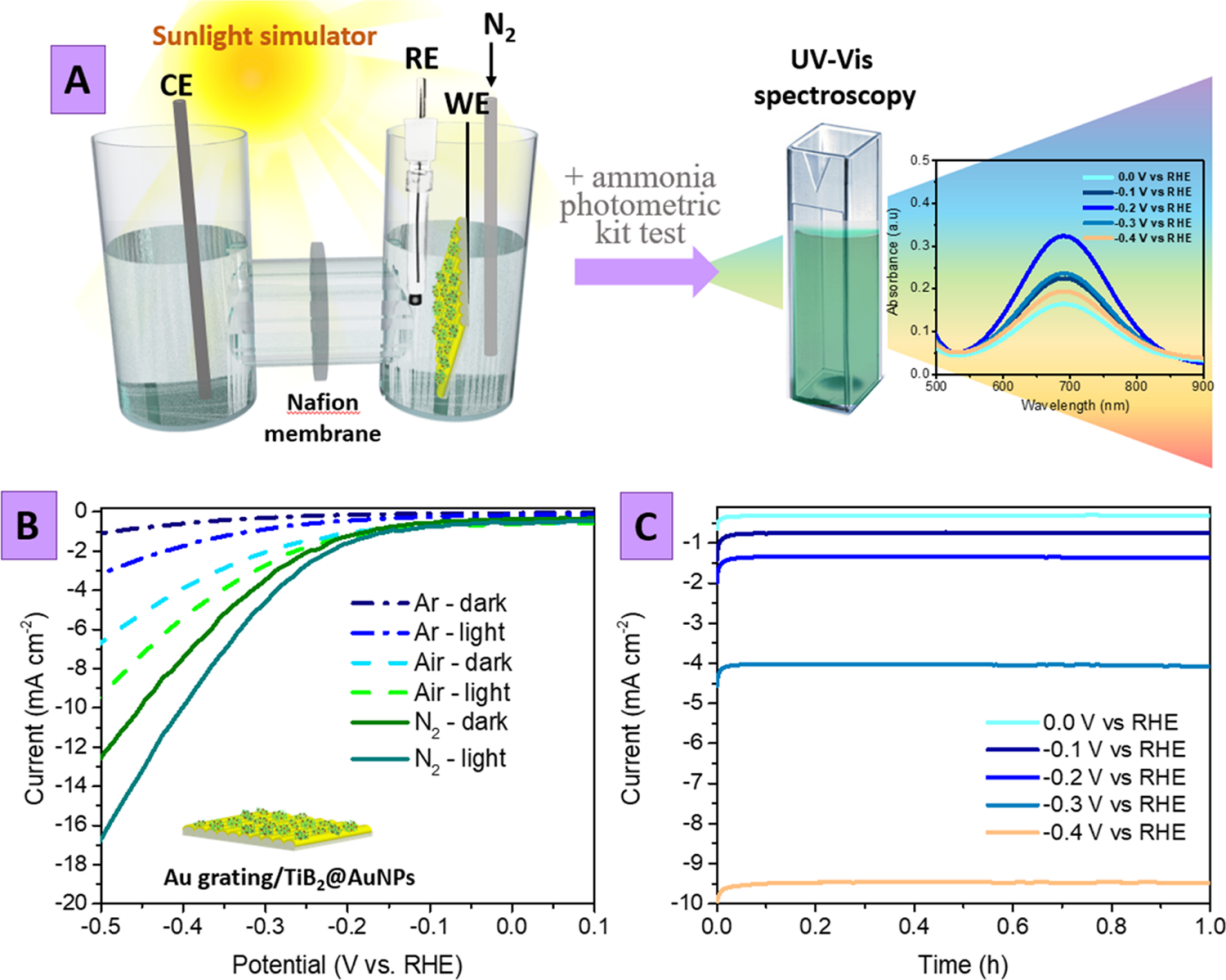
(A) Schematic illustration of the ammonium photoelectrochemical production in the H-type cell under simulated sunlight illumination; (B) LSV plots measured on the Au grating/TiB_2_@AuNP photoelectrode in the dark and under irradiation in Ar, air, and N_2_-saturated solution; (C) current densities measured in the chronoamperometry regime at various potentials and simulated sunlight illumination on the Au grating/TiB_2_@AuNP photoelectrode in N_2_-saturated solution under sunlight illumination.

In the next step, we proceed to the evaluation of the created structural efficiency in the NRR performed in the PEC regime. The obtained results are presented in Figures 3 (photoelectrochemical characterization) and 4 (ammonia production rate), revealing the electrical current density and amounts of evolved ammonia (or alternative gases; see the Control Experiments section). All PEC experiments were performed in H-type electrochemical cells separated by the Nafion 117 membrane (Figure 3A). Since the plasmon-triggered Au grating/TiB_2_@AuNPs can also efficiently participate in HER, we block this concurrent reaction by adding ionic liquid to the reaction mixture (Figures S8 and S9). The linear sweep voltammetry (LSV) curves, measured under the reaction purging with an inert Ar and with or without simulated sunlight illumination, are presented in Figure 3B. As is evident, the characteristic LSV curves, measured in the Ar-saturated solution in the dark, have a characteristic bend, located at −0.4 V (vs. RHE), which indicates that some HER proceeds anyway. The LSV curves in the Ar-saturated solution are also slightly sensitive to LSP–SPP triggering (Figure 3B), where the observation corresponds to the results of our preliminary work (such a phenomenon was also observed for pristine Au grating; Figure S10).^32^

**Figure 4:**
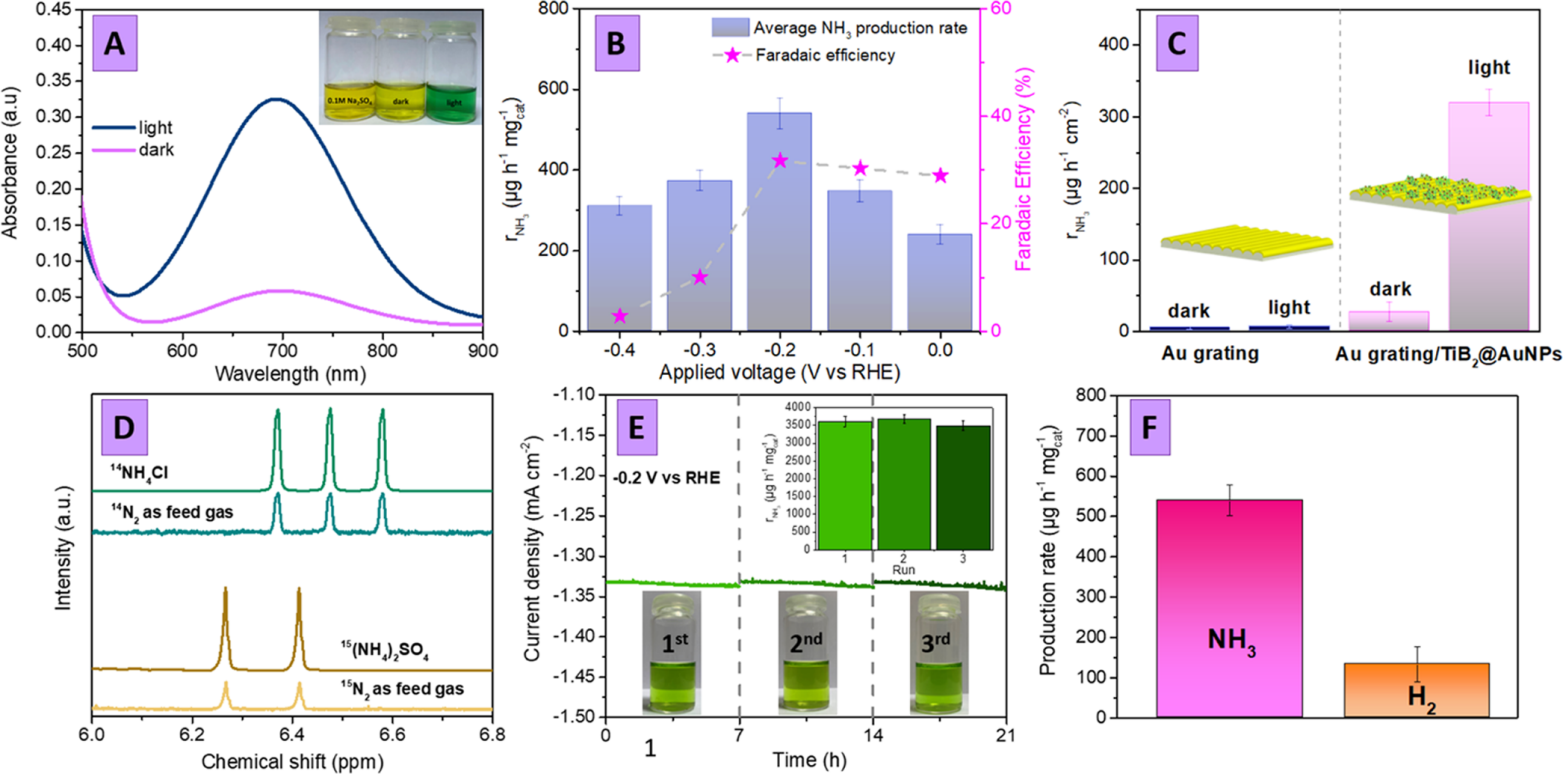
(A) UV–vis absorption spectra of ammonia photometric solutions demonstrating the difference between the characteristic band intensities obtained with the utilization of the Au grating/TiB_2_@AuNP photoelectrode (−0.2 V vs. RHE) in the dark or under simulated sunlight illumination; (B) ammonia yields and faradaic efficiencies as a function of the applied potential in PEC regime with the Au grating/TiB_2_@AuNP photoelectrode; (C) ammonia yields as a function of illumination and PEC electrodes: Au grating vs. Au grating/TiB_2_@AuNPs (−0.2 V vs. RHE); (D) ^1^H NMR spectra of isotope labeling experiments performed with the utilization of the Au grating/TiB_2_@AuNP photoelectrode and reaction mixture saturation with ^15^N_2_ or ^14^N_2_; (E) stability of Au grating/TiB_2_@AuNPs, estimated in chronoamperometry mode (3 × 7 h cycles) at −0.2 V vs. RHE and under sunlight illumination. Inserts show the color of photometric test solutions and amounts of NH_3_ produced after each cycle; (F) comparison of NH_3_ and H_2_ production rate on the Au grating/TiB_2_@AuNP surface in the PEC regime.

However, the saturation of the reaction mixture with nitrogen significantly affects the shape of the LSV curves. In this case, the electrochemical process starts at a lower potential (−0.21 V versus RHE) and proceeds with higher efficiency (the current density increases with increasing potential in a more shape manner). In this case, the LSV curve is significantly affected by illumination with simulated sunlight. In particular, the onset potential is shifted by about 0.02 V from −0.21 to −0.19 V (vs. RHE), while the 10 mA cm^–2^ current density is reached at −0.4 V vs. RHE instead at −0.45 V in dark conditions. We also performed EIS measurements, which indicate that the coupled plasmon triggering of TiB_2_ facilitates charge transfer between the electrode surface and the surrounding electrolyte, the effect being evident from the characteristic changes of the Nyquist plot induced by simulated sunlight illumination (Figure S11).

Finally, the light switching experiments, measured in chronoamperometry mode, also show that the current density immediately increases after sunlight switching ON and returns to its initial value after switching OFF (Figure S12). Results of chronoamperometry tests (also subsequently used to estimate a more suitable potential for NRR) are presented in Figure 3C. In this case, the Au grating/TiB_2_@AuNP electrode was kept at a constant potential in the N_2_-saturated solution under simulated sunlight illumination. As is evident, the initial drop in the current density is observed for all potentials used. After the current density drop, the plateau was reached with values dependent on the applied potential. It should also be noted that the observed current density is related to consumed electrons, which can participate in both nitrogen and water reduction reactions and the higher value of current is not strictly proportional to the higher amount of ammonia produced.

The amount of ammonia produced was estimated after chronoamperometry measurements using the photometric kit test and a calibration curve prepared separately (Figure S13). Typical UV–vis results are presented in Figure 4A, where the pronounced differences in the characteristic absorption band intensity indicate ammonia production, especially effective in the PEC mode under the application of external bias and simulated sunlight illumination. The amount of ammonia produced in the PEC regime as a function of applied potential is presented in Figure 4B (Figure S14 shows the corresponding UV–vis spectra). As is evident, the highest amount of ammonia was achieved in the case of −0.2 V (vs. RHE potential), despite the current density values presented in Figure 3B. So, even at −0.2 V and simulated sunlight illumination, the NRR reaction proceeds under optimal conditions, whereas a subsequent increase of applied potential supports rather the HER one. The calculation of the faradaic efficiency gives a value close to 32%, also achieved under an applied potential of −0.2 V.

### 3.3 Control Experiments

The control experiments (see Figure 4C; derived from UV–vis measurement—Figure S15), performed in the dark or without the addition of catalytically active TiB_2_ flakes on the Au grating surface (or between AuNPs and the Au grating), indicate that some ammonia is produced even under such conditions but with significantly lower yields [the ammonia yield decrease by 1.8 times in the absence of TiB_2_ flakes (Figure S16) and by 12.2 times in the absence of illumination (Figure 4C)].

To further highlight the role of coupled plasmonic hot spots, we performed several experiments where the NRR was also performed on samples without the LSP–SPP coupling (Figure S16). In particular, we used the TiB_2_ flakes deposited on Au grating (without AuNPs responsible for LSP excitation), TiB_2_@AuNPs deposited on the carbon electrode surface (without the component responsible for SPP excitation), or TiB_2_ deposited on the carbon surface (missing plasmon excitation). In all cases, we observed a significant decrease in ammonia production rates, namely, 176.2 μg h^–1^ cm^–2^ for the Au grating/TiB_2_ sample, 93.1 μg h^–1^ cm^–2^ for the TiB_2_@AuNP sample, and 63.4 μg h^–1^ cm^–2^ for the TiB_2_ sample. Therefore, from these control experiments, we can state that the LSP–SPP coupling is a key factor that ensures the high catalytic activity of the TiB_2_ flakes.

To finally confirm the N_2_ → NH_3_ chemical transition, we additionally performed the ^15^N_2_ isotopic labeling experiment (with utilization of −0.2 V vs. RHE potential and simulated sunlight illumination for 1 h) involving the reaction solution saturated with ^15^N_2_ instead of ^14^N_2_.^35,36^ The reaction mixture was analyzed using the NMR technique (Figure 4D). With the utilization of ^15^N_2_, the 1 H nuclear magnetic resonance (^1^H NMR) spectrum shows doublet coupling for ^15^NH_4_^+^, while for the ^14^N_2_ feed gas, triplet coupling for ^14^NH_4_^+^ was observed. Since we used the ^15^N_2_ with atom 98% isotope concentration, the absence of a “residual” triplet signal from 2% of ^14^N atoms can be explained by its low intensity at a noise level, which was checked by control experiments (Figure S17). These results provide the final evidence of the generation of ammonia from nitrogen delivered to the reaction mixture and exclude the influence of possible contamination from the supplied nitrogen gas or catalyst, which could lead to the appearance of false results. It should also be noted that we did not observe any significant differences in the ammonia yield and overall faradaic efficiency (Figure S18) with the use of ^14^N_2_ or ^15^N_2_ gases.^37−39^

It was also important to check the purity of the nitrogen gas because of the possible presence of impurities such as NO*_x_* species (or ^15^NH_3_ species), which can be easily reduced, providing false results in this way.^40^ This phenomenon can be especially critical in the case of ^15^N_2_ gas utilization.^41^ To assess the purity of our ^15^N_2_ tank, some steps were taken.^42^ First, a 0.1 M Na_2_SO_4_ solution was purged with Ar to remove excess ^14^N_2_. Then, approximately 200 mL of ^15^N_2_ was bubbled through the solution for 1 h. The resulting solution was then subjected to colorimetric tests to detect NH_4_^+^, NO_3_^–^, and NO_2_^–^. Using colorimetric tests, the presence of 0.06 mg L^–1^ of NH_4_^+^, 0.1 mg L^–1^ of NO_3_^–^, and 0.095 mg L^–1^ of NO_2_^–^ in the solution was found, as shown in Figure S13. Based on these results, it can be stated that the contamination of our system with NH_4_^+^ and NO_3_^–^ is negligible. However, it should be noted that NO_2_^–^ is easily converted to NH_4_^+^ and may give false positives even in the isotope-labeled test. To reduce impurities and avoid false positives, a commercial gas purifier (GateKeeper GPU EX Media Gas Purifiers) and nitrogen passage through an absorber (1 mM H_2_SO_4_) followed by deionized water were used in part of ^15^N_2_ experiments. Gas utilization was verified with/without this gas purifier, and the obtained results indicate no significant differences (Figure S19).

Finally, NMR-based measurements were also used to check the spectrophotometry tests. For this goal, an additional calibration curve was created (Figure S20) and subsequently utilized for the estimation of the ammonia yield (for an optimal case −0.2 V vs. RHE, 1 h, simulated sunlight illumination.). The results of spectrophotometry and NMR correlate well: 2.2 and 2.4 g L^–1^ of ammonia were found in the reaction mixture using these two methods.

We also perform a range of long-time stability as well as selectivity toward ammonia tests. In the case of chronoamperometry experiments (Figures 4E and S21), we did not observe any decrease in the current density during the 21 h of the utilization of Au grating/TiB_2_@AuNP samples in the PEC NRR (in particular, 7 runs for 3 h, separated by a sampling of the reaction mixture for further analysis). The amounts of ammonia produced were measured every 3 h (Figure S22), and it was found that the ammonia yield remains at the same level during all long-term experiments. Therefore, stability experiments show that the efficiency of the proposed system is conserved without any decrease during long-time utilization. Subsequently performed XPS analysis of the electrode surface (Figure S23) indicates the absence of significant changes in the Au grating/TiB_2_@AuNP surface composition but slight changes in Ti and B oxidation states (a slight reduction of B was observed too). A slight decrease of Ti and B surface concentrations was also observed after the stability tests. This decrease can be attributed to the partial detachment of those TiB_2_@AuNP nanostructures that are less tightly bound to the Au grating surface. However, the conservation of the current density and ammonia yield indicates that this part of TiB_2_@AuNPs has no significant catalytic function.

XPS measurements of the chemical composition of the used electrode surface also indicate the presence of nitrogen atoms (Figure S23). Therefore, the origin of nitrogen in ammonia produced should be additionally clarified.^38^ First, the control experiments performed with the reaction mixture bubbling with Ar do not indicate any ammonia presence (Table 1). Second, the XPS measurements performed after Au grating/TiB_2_@AuNP photoelectrode utilization in the long-term ammonia production reveal just a negligible decrease in the surface nitrogen concentration (which can rather be associated and correlated with the detachment and loss of TiB_2_@AuNP flakes from the electrode surface). Thus, based on these two observations, we can state that the origin of nitrogen in produced ammonia should be attributed to N_2_ delivered into the reaction system.

**Table 1:** NRR Rates Obtained with the Utilization Nitrogen/Argon Mixture or Atmospheric Air as a Nitrogen Source in the PEC Regime. NRR rates for a solely electrochemical or photochemical process are also included

catalyst	conditions		NH_3_ yield (μg h^–1^ mg_cat_^–1^)
Au grating/TiB_2_@AuNPs	light (PEC NRR)^a^		535.2
	PEC NRR	argon/N_2_ (10/90)	525.8
		argon/N_2_ (20/80)	502.4
		argon/N_2_ (30/70)	474.9
		argon/N_2_ (50/50)	386.3
		argon/N_2_ (70/30)	248.6
		argon/N_2_ (80/20)	144.5
		argon/N_2_ (90/10)	58.5
		argon	0
		**air**	**491.3**
	dark (electrochemical NRR)^a^		43.7
	photocatalytic experiments^a^		87.5

a N_2_-saturated solution; PEC regime: sunlight simulation, −0.2 V vs RHE, 1 h.

The proceeding of concurrent reactions—production of hydrogen and hydrazine on the Au grating/TiB_2_@AuNP photoelectrode surface—was also estimated under the previously optimized experimental conditions (−0.2 V vs. RHE, simulated sunlight illumination). In the case of hydrogen, we observe its insignificant amount (Figure 4F), which could be expected due to hydrogen evolution blocked by the addition of ionic liquid (Figures S8 and S9). The amounts of hydrazine were determined by Watt and Chrisp’s method using the calibration curve prepared separately (Figure S24). We did not detect even trace amounts of hydrazine (0.0 μg h^–1^ mg_cat_^–1^). So, based on plasmon coupling utilization, the proposed Au grating/TiB_2_@AuNs photoelectrode ensures high stability and selectivity toward ammonia in the photoelectrochemical nitrogen reduction.

### 3.4 Utilization of Air as a Source of Nitrogen

In the next step, we examine the possibility of the utilization of air as a source of nitrogen in ammonia production. The results obtained with a gradual decrease of the nitrogen concentration in the gas mixture (a mix of nitrogen and argon was used) indicate that the amount of ammonia produced gradually decreases with the decrease of nitrogen concentration (Table 1). However, even in the 1:1 gas mix, we still receive an ammonia production rate equal to 386.3 μg h^–1^ mg_cat_^–1^. The utilization of ambient air (without any purification) led to a reaction rate equal to 491.3 μg h^–1^ mg_cat_^–1^ (Table 1 and Figure S25). So, the high system efficiency reached with the utilization of plasmon coupling allows us to meet two main criteria in ammonia production: utilization of renewable energy (sunlight energy) and abundant raw material (air).

### 3.5 Photocatalytic Experiment

The control experiments carried out in the absence of light or without external bias application are also noteworthy (Table 1). In the first case, we observed a drop in the NRR rate to 43.7 μg h^–1^ mg_cat_^–1^. However, in the second case, ammonia continued to be produced efficiently, even without external charge carriers—the observed NRR constant was 87.5 μg h^–1^ mg_cat_^–1^, which is very close to the excellent results recently reported.^25^ Therefore, we can conclude that the NRR reaction is more sensitive to the absence of light (than the external bias), which once again emphasizes the key role of plasmon coupling in the efficient activation of the TiB_2_ catalytic activity.

### 3.6 Comparison with Previously Published Results and Calculation of Reaction Pathway

We also compare our results with those previously published (Table 2).^20,22,23,25,43−55^ As is evident, the best recent results are in the 1–40 μg h^–1^ mg_cat_^–1^ NRR rate range (with the utilization of an external bias in the −0.2–(−0.8) range). The corresponding faradaic efficiency ranges from 3 to 36%. The use of such rare materials as black phosphorus allows one to overperform this result and increases the rate of ammonia production to 102.4 μg h^–1^ mg_cat_^–1^,^54^ while Au-PTFE/TS for the significantly higher value of FE was reported to be 37.8%.^20^ To the best of our knowledge, the amount of ammonia produced in our case is higher compared to the best previously published cases. We are also close to the highest FE value, reported in ref (55). This result should be attributed to the contribution of plasmon coupling, which can ensure the gigantic value of the electric field achieved between the AuNP(s) and Au grating, which is exactly the place where TiB_2_ flakes are located. To explain this phenomenon, we subsequently performed a range of calculations aimed at revealing potential reaction pathways and the contribution of the plasmon triggering.

**Table 2:** Comparison of Our Results with Previously Reported Ones for Photoelectrochemical NRR

catalyst	electrolyte	applied potential (V vs. RHE)	NH_3_ yield		FE (%)	ref
Au-PTFE/TS	0.05 M H_2_SO_4_	–0.2	μg h^–1^ cm^–2^	18.9	37.8	(20)
BiVO_4_/PANI	0.1 M Li_2_SO_4_	–0.1		0.93	26.43	(22)
MoS_2_@LZO	0.1 M KOH	–0.4		10.4	2.25	(23)
CQDs/STO	0.1 M Na_2_SO_4_	–0.3		32.56	10.2	(25)
Cu_2_S-In_2_S_3_	0.2 M K_2_SO_4_	–0.6		23.7	33.25	(43)
Au/SiO_2_/Si	0.05 M K_3_PO_4_	–0.2		22.0	23.7	(44)
Cu_2_O	0.1 M KOH	0.4		7.2	20.0	(45)
CeO_2_-FeB/P	0.5 M Na_2_SO_4_	–0.12		9.54	10.1	(46)
Au NRs	0.1 M KOH	–0.4		0.54	6.0	(47)
B-doped Bi nanorolls	0.05 M H_2_SO_4_	0.48	μg h^–1^ mg_cat_^–1^	29.2	8.3	(48)
NV-g-C_3_N_5_/BiOBr	0.05 M HCl	–0.2		29.4	11.0	(49)
B-TiO_2_/CPE	0.1 M Na_2_SO_4_	–0.8		14.4	3.4	(50)
BQD/MS	1 M Li_2_SO_4_	–0.4		18.5	33.2	(51)
Cu_2_O/Ru	0.05 M H_2_SO_4_	–0.2		37.4	17.1	(52)
β-FeOOH	0.5 M LiClO_4_	–0.75		23.32	6.7	(53)
black phosphorus	0.1 M HCl	–0.4		102.4	23.3	(54)
Mo-doped WO_3_@CdS	0.5 M H_2_SO_4_	–0.3		38.99	36.72	(55)
Au grating/TiB_2_@AuNPs (sunlight simulation)	0.1 M Na_2_SO_4_	–0.2	319.4 (μg h^–1^ cm^–2^)		31.7	this work
			535.2 (μg h^–1^ mg_cat_^–1^)			

**Figure 5:**
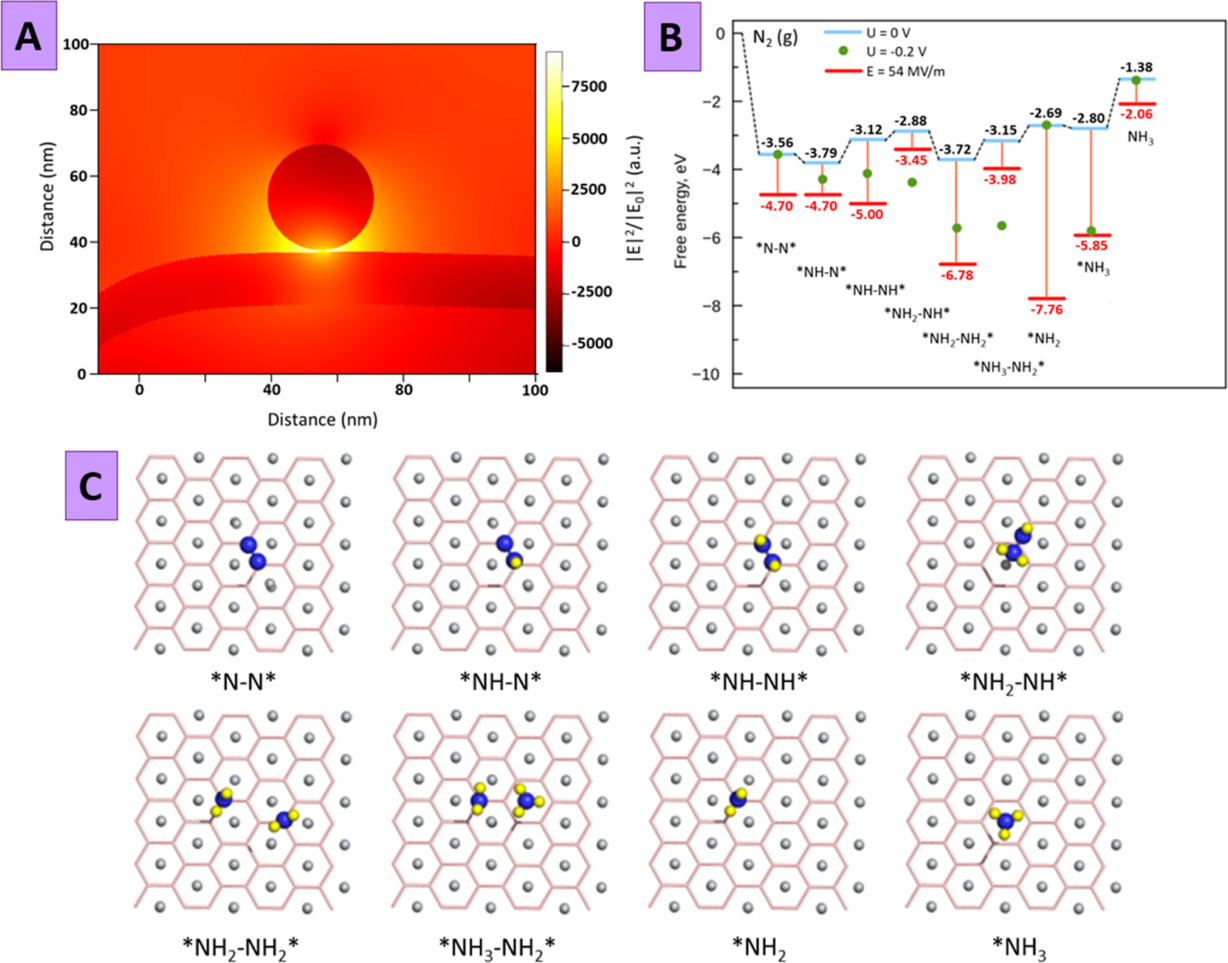
(A) FDTD-calculated distribution of the plasmon-related volumetric energy density under the illumination of the coupled AuNP–Au grating system with TiB_2_ spacer; (B) calculated free-energy diagrams for the NRR on the Ti-(001) surface under external (or zero) potential and plasmon-related electric field 54 MV m^–1^ through the enzymatic pathway; and (C) atomic structures of the corresponding intermediates.

The electronic structure of TiB_2_@AuNPs, estimated from the Mott–Schottky and Tauc plot combination, indicates that this material is suitable for the NRR position of the valence band (Figures S26 and S27 and related discussions). The results of the FDTD simulation of the plasmon-related electric field distribution reveal that the light energy is really concentrated in the TiB_2_ spacer—the space between Au grating and AuNP (Figure 5A). In this case, the preliminary delamination of TiB_2_ and the utilization of the thin flakes become critical. Particularly, the small thickness of the TiB_2_ nanostructures provides a 3–4 nm dielectric gap between the plasmon-active Au grafting and AuNP, which is a mandatory condition for the efficient plasmon coupling and energy concentration in the region between the plasmon-active structures. It is also worth noting that with an increase in the distance between plasmon-active nanostructures, their coupling can significantly weaken (Figure S28), which leads to a much less efficient energy concentration in the intermediate region (i.e., in the region of the redox-active material). In our case, the created LSP–SPP plasmonic coupling leads to a volumetric energy density increase by a factor of about 8000 (see the SI for details). In the next step, we analyzed the NRR proceeding on the TiB_2_ surface for two key cases: with and without the presence of an external electric field. In particular, we considered the NRR mechanism on the Ti-(001) surface of the TiB_2_ flake consisting of three layers (Ti–B–Ti) as the most energetically favorable,^33^ which also corresponds to concentrations observed in XRD data. In coincidence with previous studies,^33,56^ it was assumed that the nitrogen molecule is side-on-bonded on the surface, and hydrogenation occurs sequentially following the enzymatic mechanism.

In our model, we simulated the electric field in the direction of the z-axis perpendicular to the Ti-(001) plane arising during the photoelectrochemical experiment on the nanoparticle surface after irradiation with a 100 mW cm^–2^ laser (details are given in the SI). For comparison, we also performed electrochemical reduction calculations with the external applied potential (or zero potential) using the Nørskov approach^57^ with the aim to estimate free energy. Figure 5B shows the free-energy diagrams of NRR under different potential conditions with corresponding atomic geometries of nitrogen intermediates on the Ti-(001) surface as shown in Figure 5C. In general, the change in free energy starting from the binding one of a nitrogen molecule and further in the process of hydrogenation is more prominent in the case of a plasmon-related electric field of 54 MV m^–1^ than in the case of zero and external applied potentials. The N–N bond breaks in the elementary hydrogenation step of *NH_2_–NH* into *NH_2_–NH_2_*, accompanied by the release of free energy of −3.33 eV at the plasmon-related electric field against −1.12 eV when applying external bias or −0.86 eV at zero potential. Following the idea that the positively charged atomic layer of Ti is responsible for the excellent NRR activity,^33^ an electric field enhancement near the TiB_2_ flake surface (due to coupled plasmon triggering) significantly increases N_2_ affinity and promotes NRR.

## 4 Conclusions

An efficient reduction of nitrogen with the selective creation of ammonia over performing most of the previously published results was demonstrated. NRR was carried out in the photoelectrochemical regime on the surface of thin TiB_2_ nanostructures, placed in the coupled plasmon space, and subjected to nano-focused light energy. For this goal, the Au nanoparticles were prepared on the TiB_2_ surface and the created material was deposited on the surface of plasmon-active gold grating. The interaction of the gold grating surface and AuNPs with incident photons allows us to reach the gigantic subdiffraction concentration of light energy, which triggers the catalytic activity of a “sandwiched” TiB_2_ spacer. After optimization of the reaction conditions, the NRR was performed in the photoelectrochemical regime under −0.2 V vs. RHE potential and simulated sunlight illumination (with the addition of ionic liquid for hydrogen production blocking). At these conditions, we reached the 535.2 μg h^–1^ mg^–^_cat_^1^ (or 319.4 μg h^–1^ cm^–2^) ammonia rate with the “pure” nitrogen and 491.3 μg h^–1^ mg_cat_^–1^ with air as a nitrogen source. We also demonstrated the selectivity of the proposed system toward ammonia production, as well as its stability. The range of control experiments revealed the key role of coupled plasmon triggering on the TiB_2_ catalytic activity. Finally, the observed enhanced yield of ammonia was explained using the range of simulation—FDTD of the local plasmon strength and DFT studies aiming at the estimation of the plasmon contribution in NRR.

## Supporting Information Available

Data relating to the materials and sample preparation, details of sample characterization (experimental procedures and theoretical sections); TEM and HRTEM–EDX images; UV–vis and reflection spectra; AFM images; Raman spectra and SERS mapping; results of electrochemical measurements; rate of H_2_ evolution; results of XPS measurements; Tauc and Mott–Schottky plots; and calculated band structure of the photocatalyst (PDF)
