# Supplementary material for: Nitrogen Photoelectrochemical Reduction on TiB2 Surface Plasmon Coupling Allows Us to Reach Enhanced Efficiency of Ammonia Production
Source: ACS Catal. 2023 Aug 3;13(16):10916–26. doi: 10.1021/acscatal.3c03210 (PMC10442910; doi:10.1021/acscatal.3c03210)
Supplement: Supplementary file 1 — cs3c03210_si_001.pdf [file cs3c03210_si_001.pdf]

# Supporting Information for:

# Nitrogen Photoelectrochemical Reduction on $TiB_2$ Surface - Plasmon Coupling Allows to Reach Enhanced Efficiency of Ammonia Production.

A. Zabelina[a], E. Miliutina[a], J. Dedek[a], A. Trelin[a], D. Zabelin[a], R. Valiev[b], R. Ramazanov[b], V. Burtsev[a], D. Popelkova[c], M. Stastny[c], V. Svorcik[a], O. Lyutakov[a,*]

[a] *Department of Solid State Engineering, University of Chemistry and Technology, 16628 Prague, Czech Republic*

[b] *Department of Chemistry, University of Helsinki, FI-00014 Helsinki, Finland*

[c] *Institute of Inorganic Chemistry, Czech Academy of Sciences, 250 68 Husinec-Rez, Czech Republic*

---

* *Corresponding author: lyutakoo@vscht.cz*

## Experimental

### Materials

Titanium boride (titanium boride powder < 10 μm), gold (III) chloride trihydrate (≥ 99.9 %), ammonium chloride, Ammonium-$^{15}N_2$ sulfate (98 at. % of $^{15}N$), sodium nitrate (ACS reagent, ≥ 99.0 %), sodium nitrite (ACS reagent, ≥ 97.0 %), sodium sulfate (ACS reagent, ≥ 99.0 %), hydrazine hydrate ($N_2H_4$, 50-60 %), 4-(dimethylamino)benzaldehyde, hydrochloric acid (ACS reagent, 37 %), 1-butyl-1-methylpyrrolidinium tris(pentafluoroethyl) trifluorophosphate ([C4mpyr][eFAP]), deionized water, ethanol, methanol, $^{14}N_2$ gas (99.999 %) and Ar gas (99.999 %), Air (99.999 %), $^{15}N_2$ (98 at. % of $^{15}N$), ammonium photometric kit test (0.010-3.00 mg.L$^{-1}$ ($NH_4^+$), Spectroquant, Supelco, Merck Supelco), nitrate test kit (photometric 0.10–25.00 mg/L ($NO_3$-N), 0.4–110.7 mg/L $NO_3^-$, Spectroquant, Supelco, Merck) and nitrite test kit (photometric 0.002–1.00 mg/L ($NO_2$-N), 0.007–3.28 mg/L ($NO_2^-$), Spectroquant, Supelco, Merck) were purchased from Sigma-Aldrich. All chemical reagents were used as received without further purification. Au target (purity of 99.99 %) was provided by Safina. Nafion 117 membrane was purchased from Ion-Power.

### Samples preparation

***Exfoliation of $TiB_2$.*** Layered titanium diboride sheets were prepared using a high-intensity cavitation field in an ultrasonic reactor in a water/dowanol DPM mixture (UIP1000 hd, 20 kHz, 1000 W, Hielscher Ultrasonics, GmbH) in an aqueous solution. The process involved the sonication of 1.0 g of each metal diboride powder in 100 mL of deionized water under the influence of an intense

cavitation field in the ultrasonic reactor for 60 min. According to the mechanical design of the ultrasonic horn and the input power of the ultrasonic generator (1 kW) at atmospheric pressure, the output intensity was calculated to be 100 W $cm^{-2}$ at an amplitude of 80 μm. Centrifugation (3000 rpm for 10 min) was then used to remove the poorly dispersed material.

***Preparation of $TiB_2$@AuNPs composites.*** To a 4 mL aqueous suspension of $TiB_2$ flakes (1 g in 100 mL) was added 12 mL of $HAuCl_4$ (0.1 mM). The mixture was sonicated for 30 min. The resulting composite mixture was then purified three times by centrifugation, washing with methanol, re-dispersion, and subsequent dispersion in methanol.

***Au grating preparation***. The periodic surface of the DVD with a pattern area of 0.7x2 $cm^2$ was coated with gold film about 30 nm thick by vacuum sputtering (DC Ar plasma, gas purity of 99.995 %, gas pressure of 4 Pa, discharge power of 7.5 W, sputtering time 300 s).

***Deposition of $TiB_2$@AuNPs on Au grating.*** $TiB_2$@AuNPs were deposited on the surface of an Au grating using an improved technique, resulting in a thin and homogenous coating of Au grating on $TiB_2$@AuNPs (the prepared samples are referred to as Au grating/$TiB_2$@AuNPs). The optimization was carried out in accordance with the approach described in [1], and as a final step, we utilized spin-coating deposition from a $TiB_2$@AuNPs solution in methanol (1.1 mg.$mL^{-1}$) at 500 rpm.

**Measurement Techniques**

The peak force AFM measurements were conducted using the Icon (Brucker) microscope. The HRTEM-EDX mapping was performed using Jeol 2200 FS microscope (Jeol, Japan). TEM images of $TiB_2$ and $TiB_2$@AuNPs flakes were obtained with the help of a JEOL JEM-1010 transmission electron microscope with a SIS MegaView III digital camera. SEM-EDX photos and maps were obtained on Lyra3 GMU (Tescan, CR) microscope with an accelerating voltage of 2 kV.

The X-ray photoelectron spectroscopy (XPS) was performed using a Thermo Fisher Scientific XPS NEXSA spectrometer with a monochromated Al K Alpha X-ray source working at 1486.6 eV.

X-Ray diffraction measurements were carried out using the Empyrean, Malvern Panalytical diffractometer with Cu $K_{<\alpha>}$ radiation source in $2\theta$-$\theta$ diffraction mode, the Bragg-Brentano geometry.

Raman spectra were collected using a Thermo Scientific DXR Raman microscope equipped with a 532 nm excitation wavelength. Raman mapping was performed across a surface area of 1.5×0.7 $mm^2$, having 70×40 points spaced by a gap of 0.025 mm.

UV–Vis absorbance spectra of the samples were measured using a Lambda 25 spectrometer (PerkinElmer, USA). Reflection spectra of the samples were obtained by using a HR2000 (Ocean Optics) spectrometer using the AvaLight-DHS light source (Avantes).

$^1H$ NMR were recorded on Bruker Avance III™ (500 MHz) spectrometer. The rate of $H_2$ evolution was estimated by GC-7920 (Agilent) gas chromatography system.

**Electrochemical and photoelectrochemical nitrogen reduction**

Electrochemical measurements were carried out using a Palm Sens 4 potentiostat (Palm Instruments, Netherlands) controlled by the PSTrace 5.9 program using a three-electrode two-compartment electrolytic cell (H-type), which was separated by a Nafion 117 membrane. Before experiments, the Nafion membrane was immersed in 3 wt. % $H_2O_2$ solution at 90 $^oC$ for one h. Then it was cleaned in deionized water, boiled in 5 wt. % $H_2SO_4$ water solution, and cleaned again in deionized water for 2 h. The Au grating and Au grating/$TiB_2$@AuNPs samples were used as the working electrode (active surface area - 0.7×0.6 $cm^2$). An Ag/AgCl (sat. with 3M KCl) electrode (BVT Technologies, CZ), placed in the same part of the cell as the working electrode, was used as a reference electrode. Platinum Wire electrode (BASi, USA) was used as a counter electrode (placed at another part of H-cell). All photoelectrocatalytic $N_2$ reduction experiments were carried out in chronoamperometric mode in 0.1 M $Na_2SO_4$ mixture solution containing 20 wt. % ([C4mpyr][eFAP]), which was purged with different gases ($N_2$, Ar, Air, and mixture of Ar/$N_2$) for 30 min before the experiment and then continuously purged with nitrogen during experiments. Moreover, electrochemical measurements were performed without and with the illumination of the sample's surface using the solar simulator (Solar Simulator SciSun-300, Class AAA, and the intensity on the sample surface were adjusted to 100 mW $cm^{-2}$). After measurements, all potentials were converted into the reversible hydrogen electrode (RHE) potential. All experiments, except the stability tests, were replicated five times and calculated values of standard deviation were subsequently used as error bars.

**Photocatalytic nitrogen reduction**

The photocatalytic (PC) nitrogen reduction was conducted at ambient temperature and pressure. A solar simulator (Solar Simulator SciSun-300, Class AAA, and the intensity on a sample surface was adjusted to 100 mW $cm^{-2}$) was used as an artificial source of sunlight. The 0.1 M $Na_2SO_4$ mixture solution containing 20 wt. % ([C4mpyr][eFAP]) was purged with "pure" nitrogen for 30 min before the experiment and then continuously purged with nitrogen during the PC experiment. The Au grating/$TiB_2$@AuNPs was immersed in a clear glass beaker filled with electrolyte, and the unit was illuminated for one hour using a simulator. At the end of the experiment, the sample was removed, and the reaction solution was analyzed by a photometric kit test.

**Quantification of $NH_3$**

The $NH_3$ produced was quantitatively determined by the ammonia photometric kit. According to the procedure described in the ammonia test documents, the following steps were performed: 5 mL of the reaction solution (after electrochemical measures) was mixed with 0.6 mL of reagent #1 (containing sodium hydroxide). Then some amount of reagent #2 (containing thymol) was added to the resulting solution and shaken vigorously until the reagent was completely dissolved. After 5 min, reagent #3 (containing 2-propanol) was added to the reaction solution and stirred. The resultant solution was left to stand for 5 min at room temperature and then analyzed by UV-Vis absorption spectroscopy. For the creation of the calibration curve, the known concentration of $NH_4Cl$ was added to 0.1 M $Na_2SO_4$ and 0.1 NaOH (to introduce the similar values of pH for "real" and calibration solutions) mixture solution containing 20 wt. % ([C4mpyr][eFAP]) and analyzed by the method described above. The absorbance intensity at ~ 692 nm was utilized to estimate the yield of ammonia based on the standard curve.

The $NH_3$ yields (as a function of catalyst loafing or electrode surface area) were calculated by the equations 1:

$$NH_3 \text{ yield } = \frac{C_{NH_3} \cdot V}{m_{cat} \cdot t} \text{ or } NH_3 \text{ yield } = \frac{C_{NH_3} \cdot V}{A \cdot t}, \quad (1)$$

where $C_{NH_3}$ is the total amount of $NH_3$ (measured by photometric test), $V$ is the volume of the electrolyte, $m_{cat}$ is the mass of the catalyst, $t$ is the reaction time, and $A$ is the geometric area of the photocathode. Hydrazine yield was calculated using the same equation (see control experiment section). All experiments, except the stability tests, were repeated five times.

In the case of NMR-based quantitative ammonia determination, the measurements were carried out on a Bruker 600 Avance$^{III}$ (optimized parameters: pulse sequence zgpr; number of scans 512). For creation of calibration curve the series of $NH_4Cl$ solutions with 1.5–15 mg/L concentration range were prepared. A typical approach involved mixing 125 L of the standard solution with 50 L of 4 M $H_2SO_4$ in DMSO-d6 and 750 L of DMSO-d6 before doing an instantaneous analysis, according to previously reported procedure.[2]

**Impact of $N_2$ gas(es) impurity**

Nitrate ($NO_3^-$) and nitrite ($NO_2^-$) concentrations were quantitatively determined by the nitrate and nitrite photometric kit tests, respectively.

The following steps were taken according to the instructions described in the nitrate photometric kit test document: after purging with $^{15}N_2$ (refer to section 3.3 Control experiments), 0.5 mL of 0.1 M $Na_2SO_4$ solution was added to 4.0 mL of reagent #1, which contains sulfuric and

phosphoric acids. The resulting solution was subsequently mixed with 0.5 mL of reagent #2, which contains 2,6-dimethylphenol, and left to stand for 10 min at room temperature before being analyzed by UV-Vis spectroscopy analysis. The absorbance intensity at ~ 507 nm was utilized to estimate the yield of nitrate based on the standard curve.

According to the procedure described in the nitrite photometric kit test document, the following steps were performed: some amount of reagent #1 (containing sulfanilic acid) was added to 5 mL 0.1 M $Na_2SO_4$ solution after purging with $^{15}N_2$ (refer to section 3.3 Control experiments) and shaken vigorously until the reagent was completely dissolved. The resulting mixture was left to stand for 10 min and then analyzed by UV-Vis spectroscopy. The absorbance intensity at ~ 540 nm was utilized to estimate the yield of nitrite based on the standard curve.

For the creation of the calibration curves, the known concentrations of $NaNO_3$ or $NaNO_2$ were added to 0.1 M $Na_2SO_4$ solution and analyzed according to the procedure described above.

**Stability test of Au grating/$TiB_2$@AuNPs photoelectrode in NRR**

Chronoamperometry (CA) was used to evaluate the stability of the sample activity. CA curves were obtained under continuous sunlight illumination and continuous inert $N_2$ purging of the reaction solution. The same electrochemical cell, artificial sunlight source, and electrolyte were used as in the previous PEC experiments. Three stability cycles of 7 h each and seven stability cycles of 3 h each were carried out. After each cycle, the reaction mixture was diluted (due to the detection limit of the photometric test) seven or three times respectively and then analyzed by the photometric test. The electrochemical cell was filled with a new reaction solution, and the experiment was repeated again with the same Au grating/$TiB_2$@AuNPs photoelectrode. Stability tests were repeated 3 times (presented results represent averaged values of current density and ammonia produced, the standard deviation was used for error bars creation in the last case).

**Control experiments**

***Detection of hydrazine.*** The yield of $N_2H_4$ was determined by Watt and Chrisp method. The photometric solution was prepared by dissolving para-(dimethylamino)benzaldehyde (8 g) in a mixture of HCl (40 mL) and ethanol (400 mL). 5 mL of the reaction solution after the NRR experiment was taken from the H-type cell, added to 5 mL of the prepared photometric solution, stirred 15 min at 25 °C and subjected to UV-Vis absorption analysis. $N_2H_4$ solutions with known concentrations (in 0.1 M $Na_2SO_4$, containing 20 wt. % ([C4mpyr][eFAP])) were taken as calibration standards. The absorbance value at ~ 462 nm was used to create the calibration curve.

***Isotope labeling experiment.*** The electrolyte (0.1 M $Na_2SO_4$ water solution, containing 20 wt. % ([C4mpyr][eFAP]), was purged for 30 min with $^{14}N_2$ and $^{15}N_2$ as feeding gases. The NRR experiments were performed in the PEC regime at -0.2 V (vs. RHE) potential and simulated sunlight illumination for 7 h. Subsequently, 20 mL of the electrolyte solution was concentrated into 2 mL. Then, concentrated solutions (1 mL) were mixed with HCl (1 mL, 0.1 M) and DMSO-$d_6$ solutions (0.2 mL) and used for subsequent $^1H$ NMR measurements. Aqueous solutions of $NH_4Cl$ and $(^{15}NH_4)_2SO_4$ were used as standards.

***Estimation of the impact of coupled plasmon triggering experiment.*** Control experiments were carried out using the following samples: (i) - pristine $TiB_2$ flakes, (ii) - $TiB_2$@AuNPs structures deposited on glass electrodes, and (iii) - pristine $TiB_2$ flakes deposited on Au grating. Experiments were performed in PEC mode (-0.2 V vs. RHE and simulated sunlight illumination). NRR efficiency was estimated using the photometric kit test or chronoamperometry measurements.

## Calculation of $TiB_2$@AuNPs electronic structure

Calculation of the valence and conductive bands gap position of $TiB_2$@AuNPs was performed using the combination of Tauc (created from UV-Vis measurements) and Mott–Schottky (created from EIS measurements) plots (see related discussion below).

## Finite-Difference Time-Domain simulation

The simulation was carried out using MEEP software, which employs the finite-difference time-domain (FDTD) technique. The simulated system consists of a gold grating on a polymer substrate (AFM-measured profile, grating shape) with metal nanoparticles at the "top" of the grating separated by $TiB_2$ flakes. To ensure convergence, the simulation was carried out at a resolution of 2000 px/μm. A broadband Gaussian source was used to stimulate the simulated cell. Following the completion of the time-domain simulation, the data were converted to the frequency domain using the FFT technique [3].

## Calculation of free energy profile

Estimation of the electric field on the nanoparticle surface after irradiation with a 100 mW $cm^{-2}$ laser was performed according following prerequisites:

Let us assume that the laser radiation flux density:

$$I = \omega \cdot c \; (I = 100\frac{mW}{cm^{-2}}), \quad (1)$$

where $\omega$ is the volumetric energy density, $c$ is the speed of light $\omega = \varepsilon\varepsilon_0 E^2$.

The volumetric energy density near the surface of the particle increased 8000 times, then

$$\frac{\omega}{\omega_0} = \frac{E^2}{E_0^2} = 8000, \qquad E = \sqrt{\frac{10^3}{3 \cdot 10^8 \cdot 8.9 \cdot 10^{-12}} \cdot 8000} = 54 \cdot \frac{10^3 V}{m} = 5.4 \cdot 10^{-6}\, V/Å. \qquad (2)$$

To calculate the free energy profile of the reduction of the $N_2$ molecule, we used the computational hydrogen electrode method (CHE).

$$\Delta G = \Delta E + \Delta E(ZPE) - T\Delta S + n_H U, \qquad (3)$$

where $\Delta E$ is the adsorption energy, $\Delta E(ZPE)$ is the zero point energy difference, and $T\Delta S$ is the entropy difference between the gas phase and adsorbed state. $n_H U$ – applied potential energy, where $n_H$ is the number of transferred charge, and $U$ is the electrode potential. For adsorbates, $ZPE$ and $S$ are determined by vibrational frequencies calculations. For molecules, those are taken from the NIST database [4].

**Density functional calculation of plasmon-assisted NRR**

The DFT calculations were carried out with the CP2K package.[5]The DFT calculations used the combination of the Gaussian and plane-wave (GPW)[6] scheme for electronic density representation, double-ζ valence plus polarization (DZVP) basis sets of the MOLOPT[7] type to describe the valence electrons and norm-conserving Goedecker-Teter-Hutter (GTH)[8–10] pseudopotentials to approximate the core electrons. The generalized gradient approximation (GGA) of the Perdew−Burke−Ernzerhof (PBE) functional[11] was used to describe the exchange-correlation interactions. DFT-D3 method[12] was adopted to consider the van der Waals (vdW) interaction. For the $TiB_2$ flake description, we used the Ti-(001) surface covering of three-layer (Ti-B-Ti) bulk structures placed in 15.76×10×15.19 $Å^3$ size box with periodic boundary conditions in the XY plane.

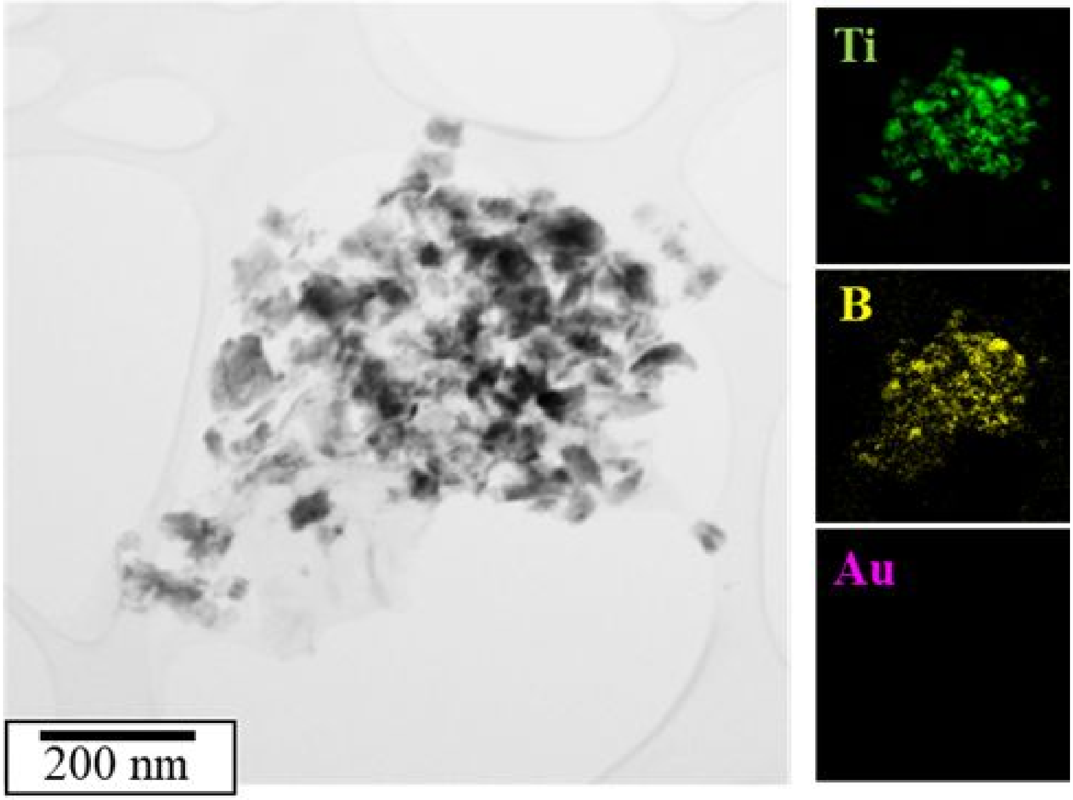


**Figure S1** TEM image with corresponding EDX mapping of pristine $TiB_2$ flakes.

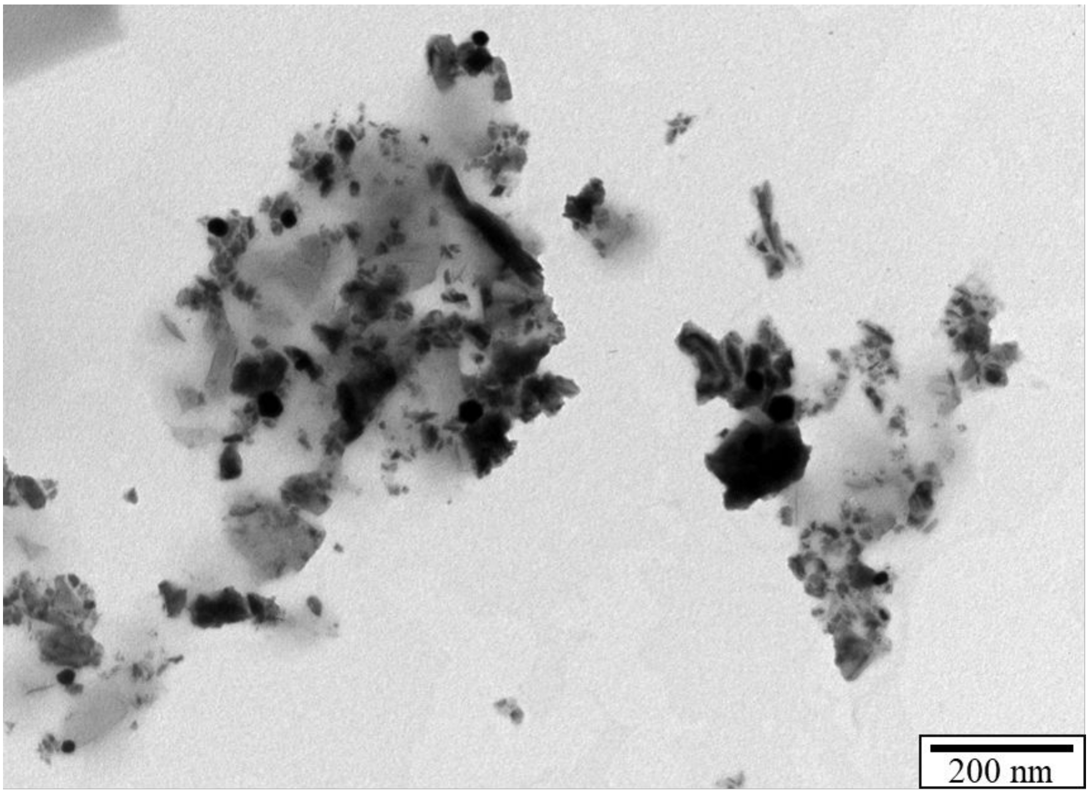


**Figure S2** TEM image of $TiB_2$@AuNPs flakes.

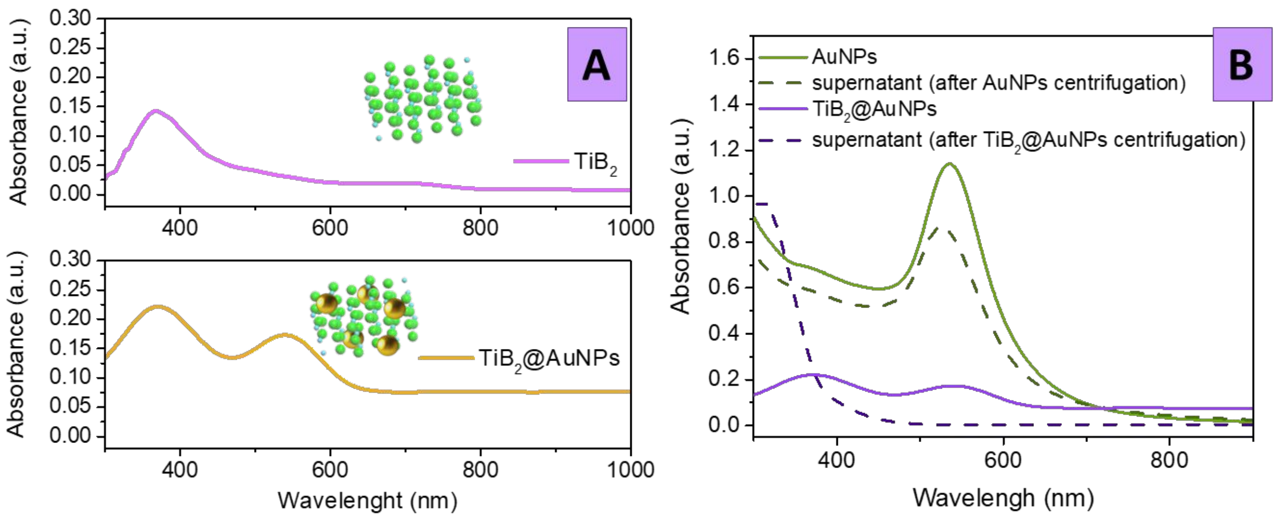


**Figure S3** UV-Vis spectra of: (A) – $TiB_2$ and $TiB_2$@AuNPs flakes, (B) – UV-Vis spectra of $TiB_2$@AuNPs and separately prepared AuNPs before and after "mild" centrifugation (7800 rmp, 20 min).

***Figs. S2, S3 – related discussion (appearance of AuNPs)***

To check formation of all AuNPs on the $TiB_2$ surface, and possible overspending of relatively expensive gold, we carried out two control measurements. First, the TEM results (Figure S2) showed that all features characteristic for AuNPs (visible as dark regions) lie in the $TiB_2$ spatial region ($TiB_2$, which is more transparent to electrons and visible as grey regions). Next, we performed precipitation of AuNPs and $TiB_2$@AuNPs suspension under mild conditions (7800 rpm and 20 min) and subjected the remaining supernatants to UV-Vis absorption measurements (Figure S3). In the case of the control sample (AuNPs suspension), precipitation under mild conditions did not lead to complete sedimentation of AuNPs and their characteristic bands remained visible. In the case of $TiB_2$@AuNPs, UV-Vis spectroscopy showed the absence of previously clearly visible absorption bands (including the characteristic band of plasmon absorption), which indicates the complete sedimentation of the "heavier" structures (i.e., $TiB_2$@AuNPs flakes) and the absence of "free" AuNPs, which, according to the control experiment, should stay in solution.

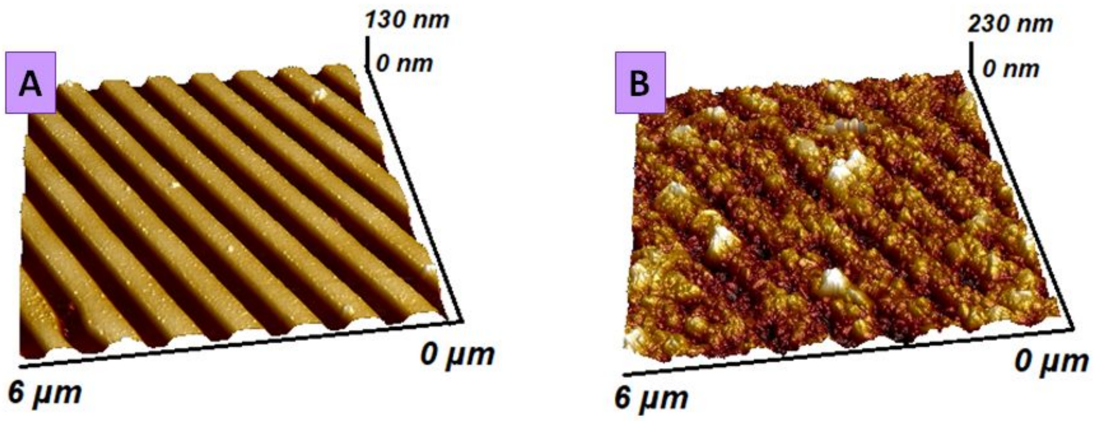


**Figure S4** AFM image of the surface morphology of Au grating before (A) and after $TiB_2$ flakes (B).

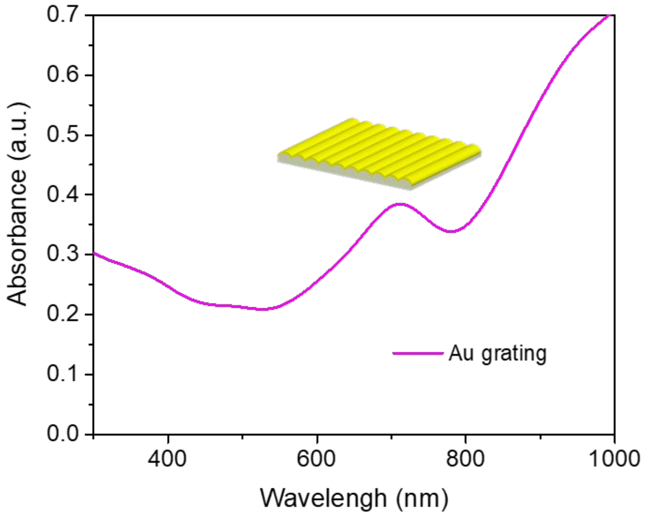


**Figure S5** Characteristic UV-Vis spectrum of pristine Au grating.

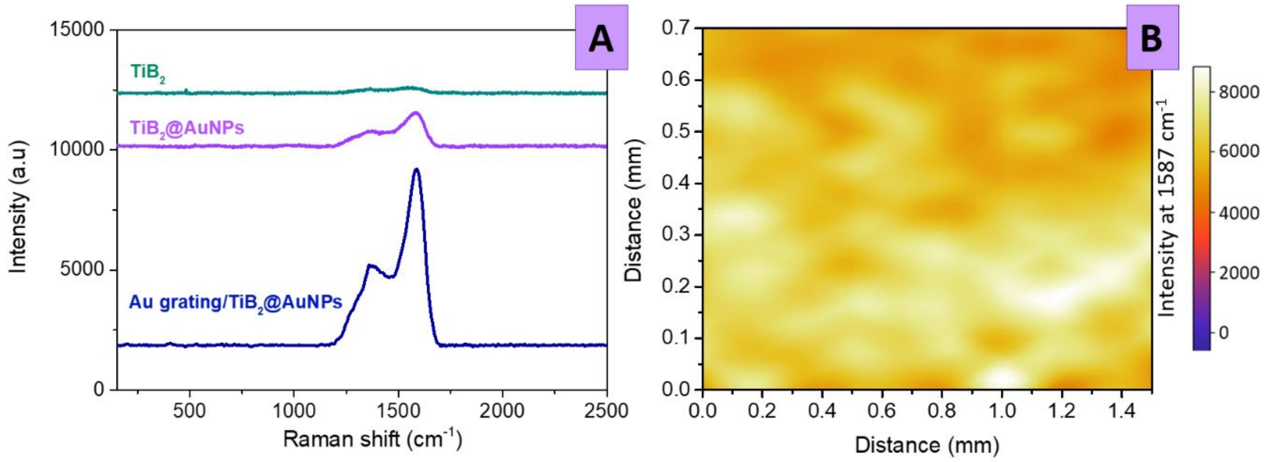


**Figure S6** (A) – Raman spectra of pristine $TiB_2$ and SERS spectra of $TiB_2$@AuNPs, measured before and after the deposition on Au grating. (B) – SERS mapping of $TiB_2$@AuNPs distribution on Au grating surface (spatial distribution of the characteristic $TiB_2$ peak at 1587 $cm^{-1}$).

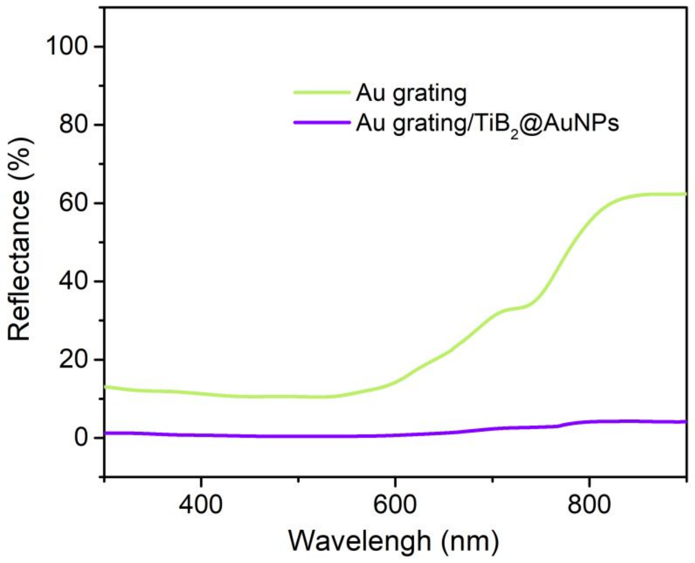


**Figure S7** Reflection spectra of pristine Au grating and Au grating/$TiB_2$@AuNPs.

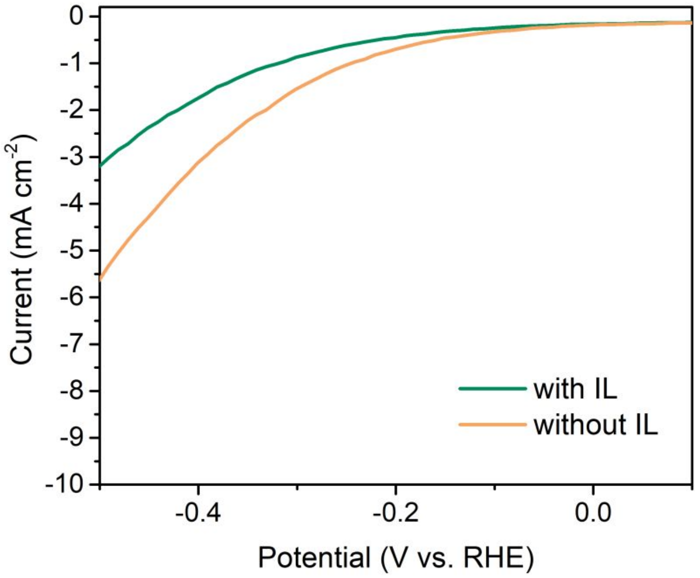


**Figure S8** LSV curves measured in PEC regime with Au grating/$TiB_2$@AuNPs as a working electrode and simulated sunlight illumination with and without the addition of ionic liquid (IL) (Ar-saturated solution).

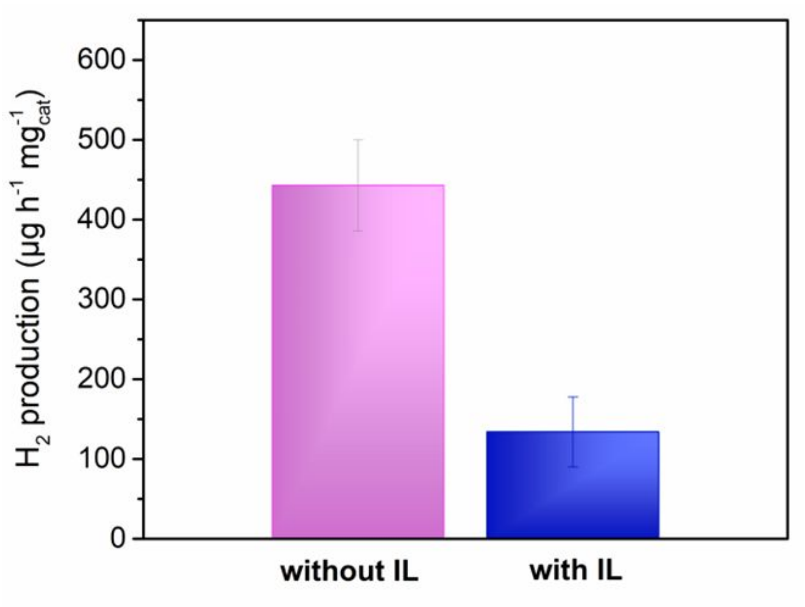


**Figure S9** Rate of $H_2$ evolution on Au grating/$TiB_2$@AuNPs electrode surface as a function of addition of ionic liquid (H-type electrochemical cell, three-electrode system, sunlight illumination, applied potential -0.2V vs. RHE, $N_2$-saturated solution).

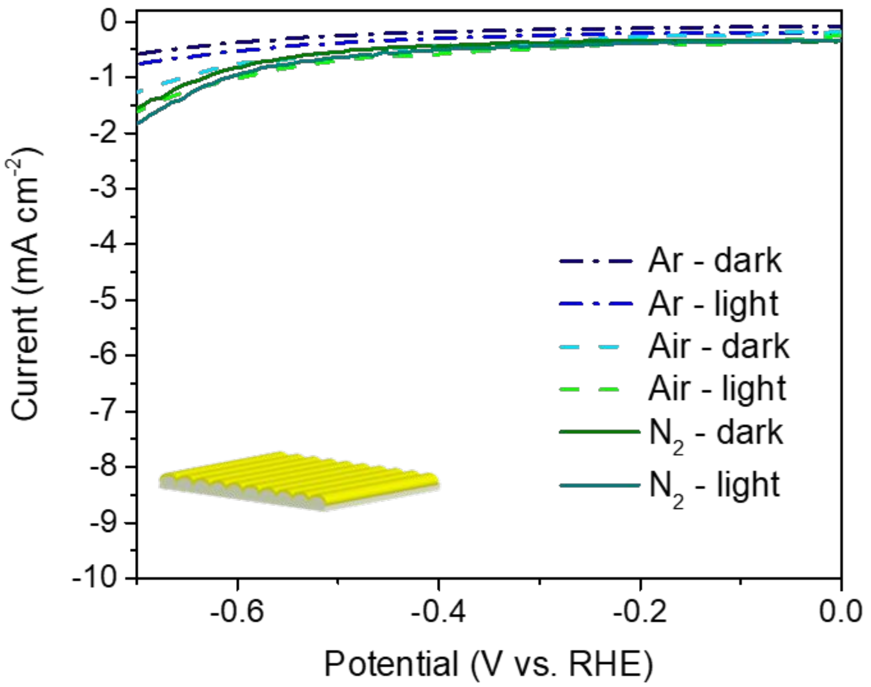


**Figure S10** LSV plots, measured in a three-electrode system (H-type electrochemical cell) with Au grating as the working electrode (electrolyte saturation with $N_2$ or Ar, simulated sunlight illumination switched ON or switched OFF).

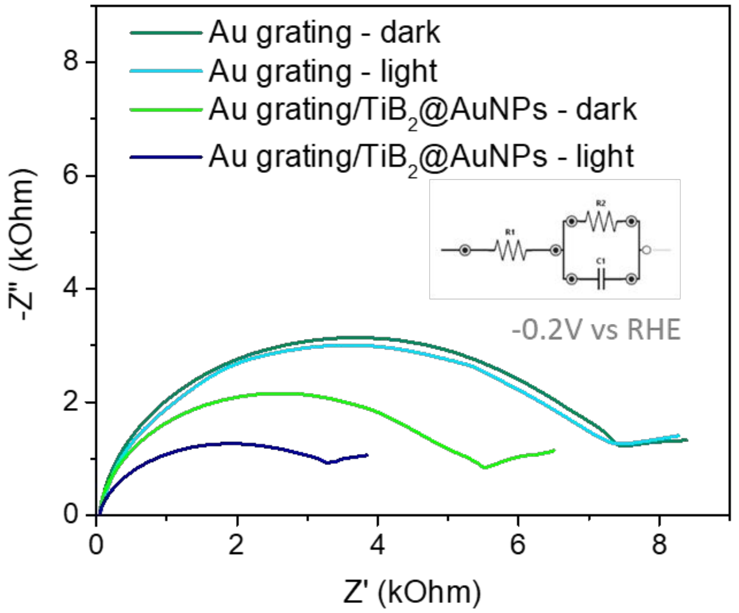


**Figure S11** Nyquist plots of the Au grating and Au grating/$TiB_2$@AuNPs in the dark and under light illumination (H-type electrochemical cell, three-electrode system, light illumination switched ON or switched OFF, applied potential -0.2V vs. RHE, $N_2$-saturated solution).

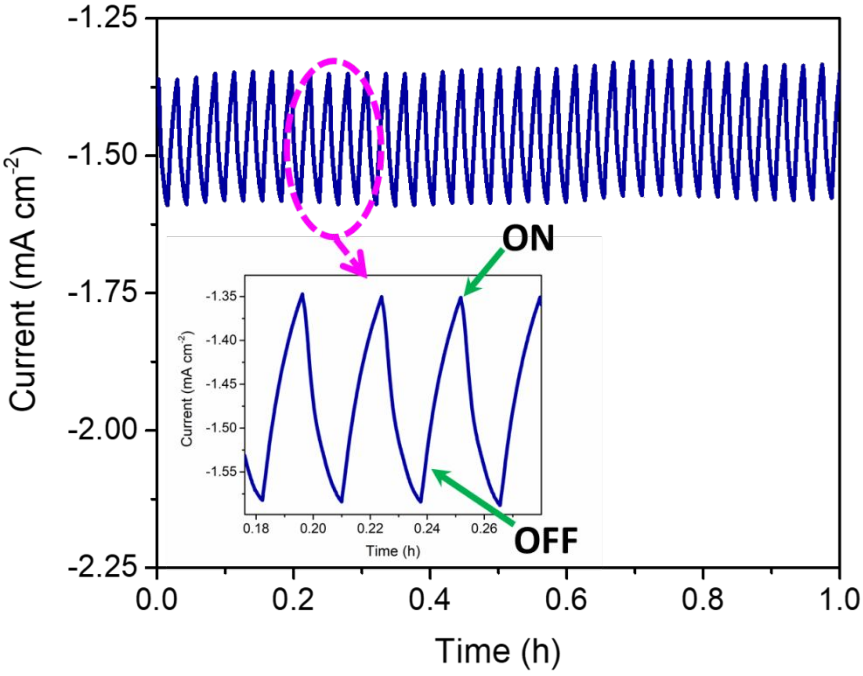


**Figure S12** Chronoamperometry characterization of Au grating/$TiB_2$@AuNPs, measured under repeated ON/OFF switching of simulated sunlight illumination in $N_2$ saturated solution at applied potential -0.2V vs. RHE.

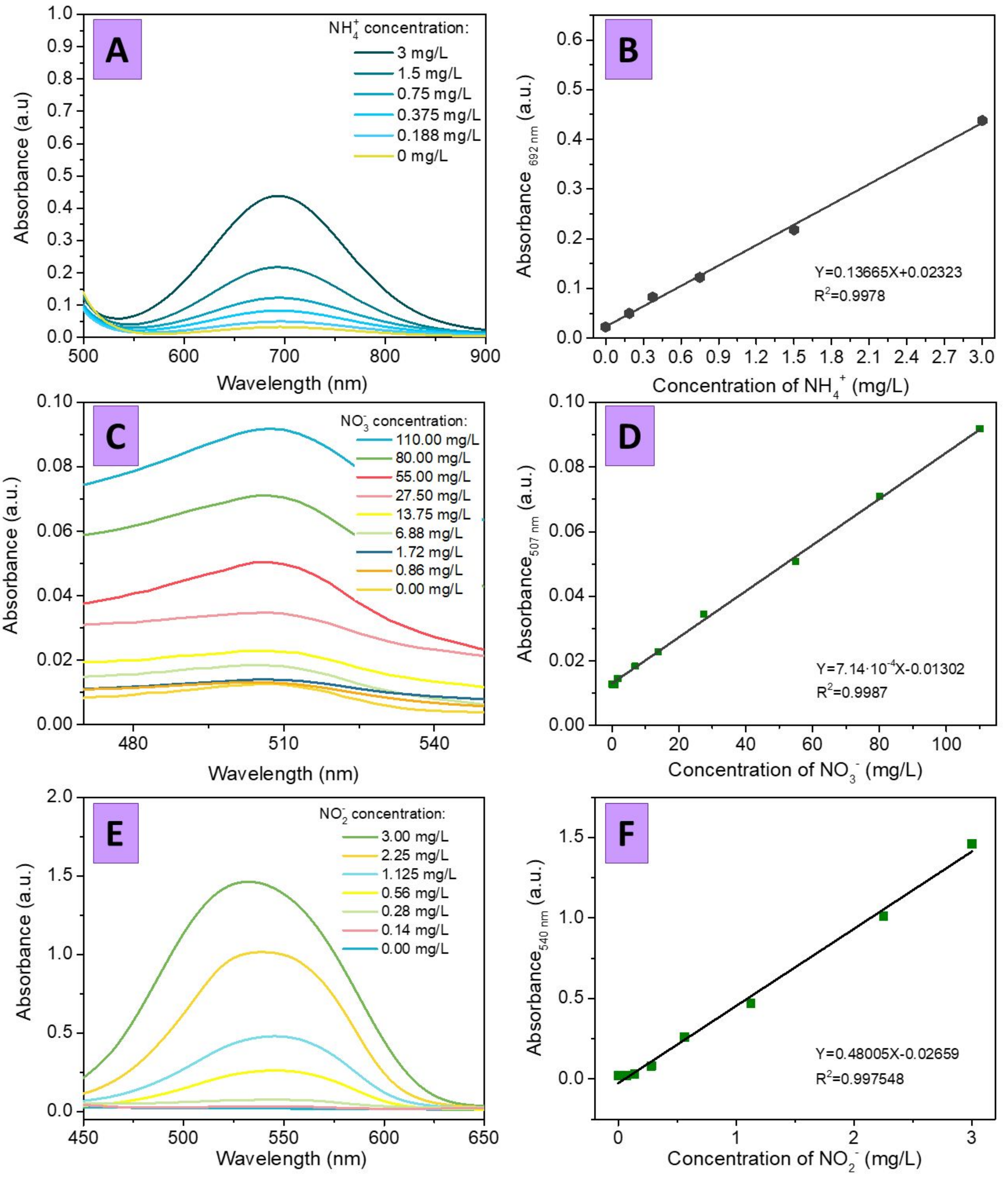


**Figure S13** UV-Vis absorption spectra of (A) – ammonia, (C) – nitrate and (E) – nitrite photometric kit tests after the addition of various amounts of $NH_4Cl$, $NaNO_3$ and $NaNO_2$ respectively. Calibration curves, calculated from UV-Vis absorption spectra and subsequently used to determine production of (B) – $NH_4^+$, (D) – $NO_3^-$ and (F) – $NO_2^-$.

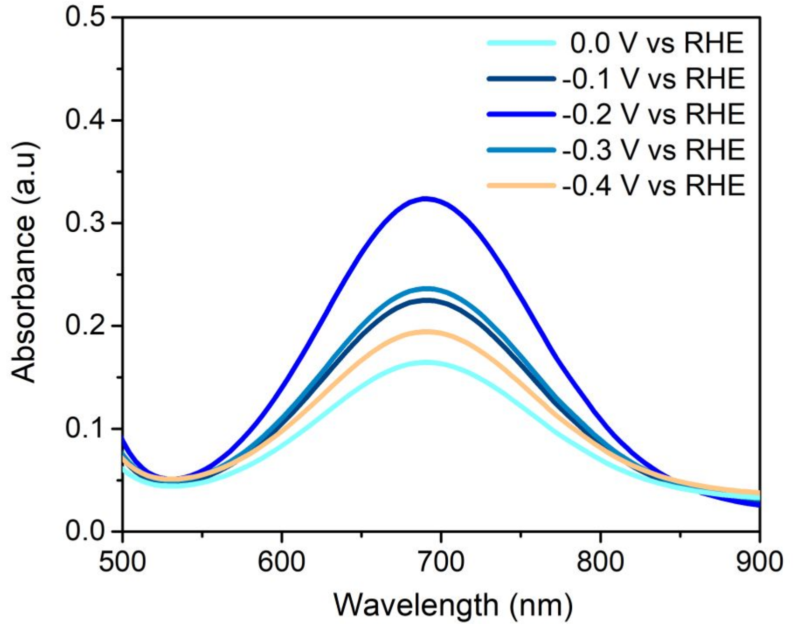


**Figure S14** UV-Vis absorption spectra of the photometric solution reveal the ammonia formation on Au grating/$TiB_2$@AuNPs surface in a chronoamperometry mode as a function of applied potential.

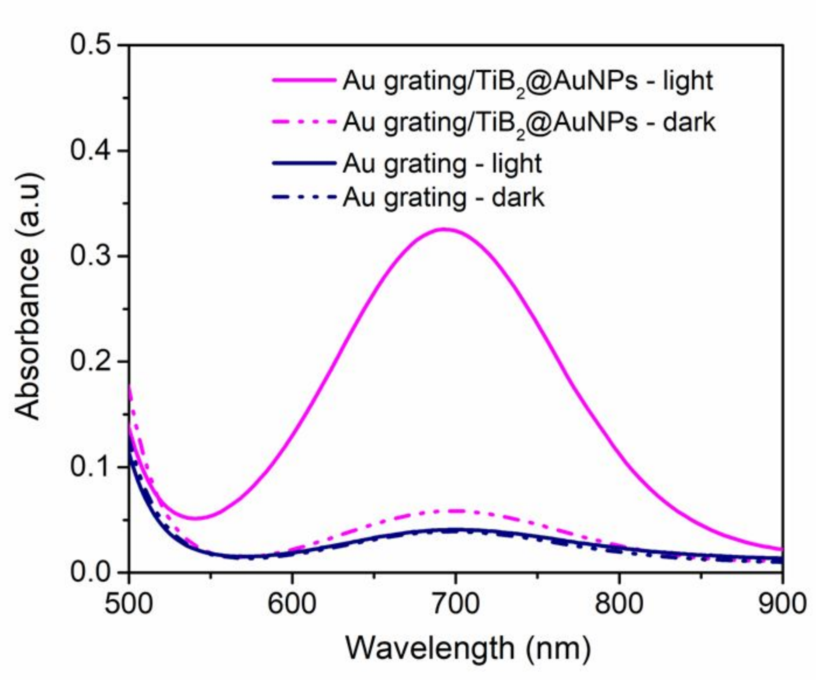


**Figure S15** UV-Vis absorption spectra of photometric solutions – revealing of the ammonia formation on Au grating/$TiB_2$@AuNPs and Au grating surfaces in a chronoamperometry mode under simulated sunlight illumination or in the dark.

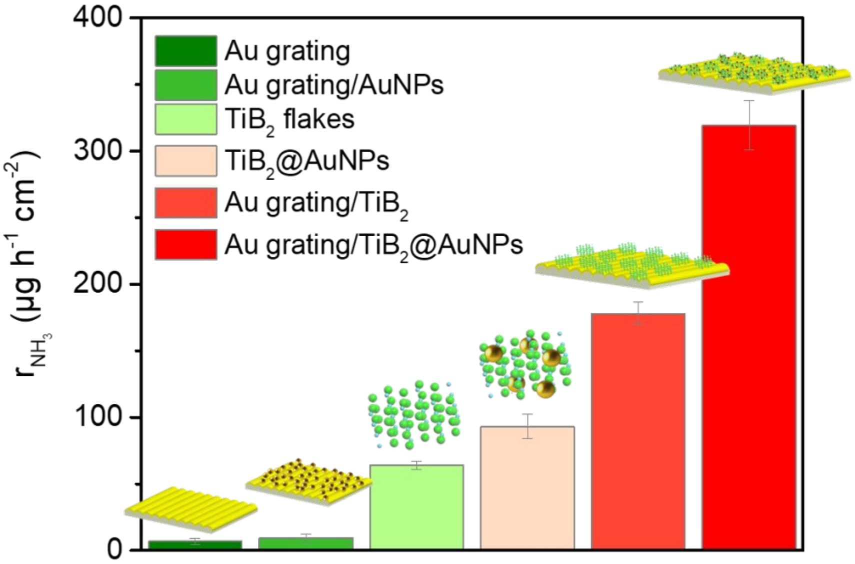


**Figure S16** Control experiments – the impact of the absence of coupled plasmon triggering of $TiB_2$ catalytic activity on $NH_3$ production (H-type electrochemical cell, three-electrode system, sunlight illumination, applied potential -0.2V vs. RHE, $N_2$-saturated solution).

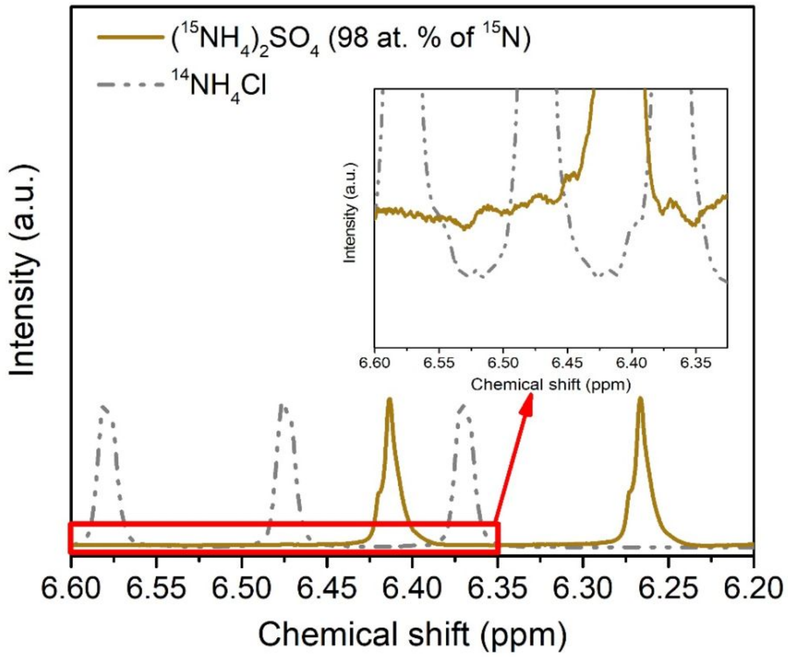


**Figure S17** Control NMR measurement of $(^{15}NH_4)_2SO_4$ – the chemical shift area, attributed to peaks from $^{14}N$ "impurities" is magnified in an insert.

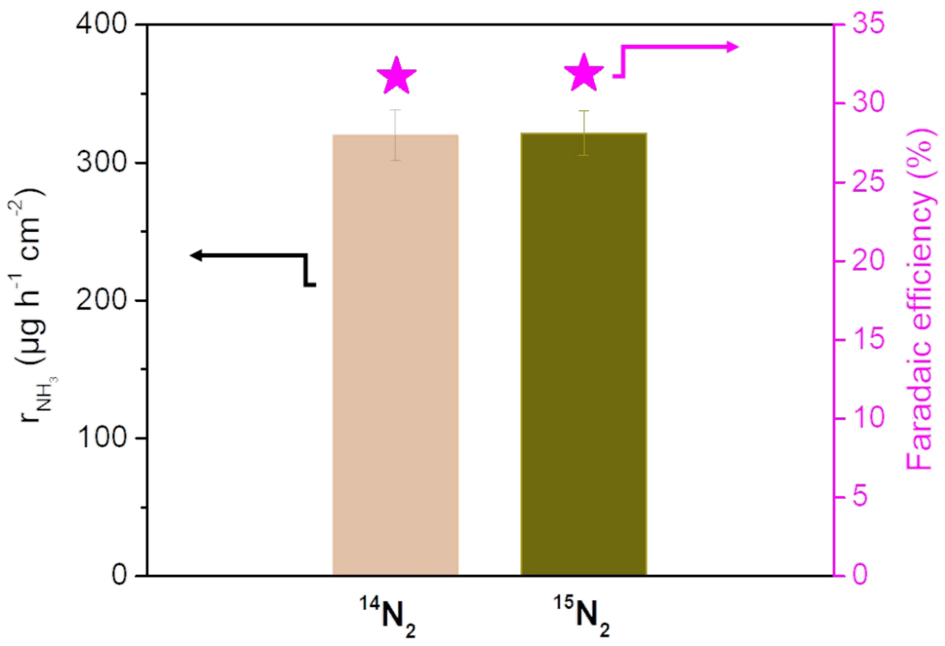


**Figure S18** Comparison of non-isotopic and isotopic ammonia yield rate and Faradaic efficiency quantified by photometric kit test.

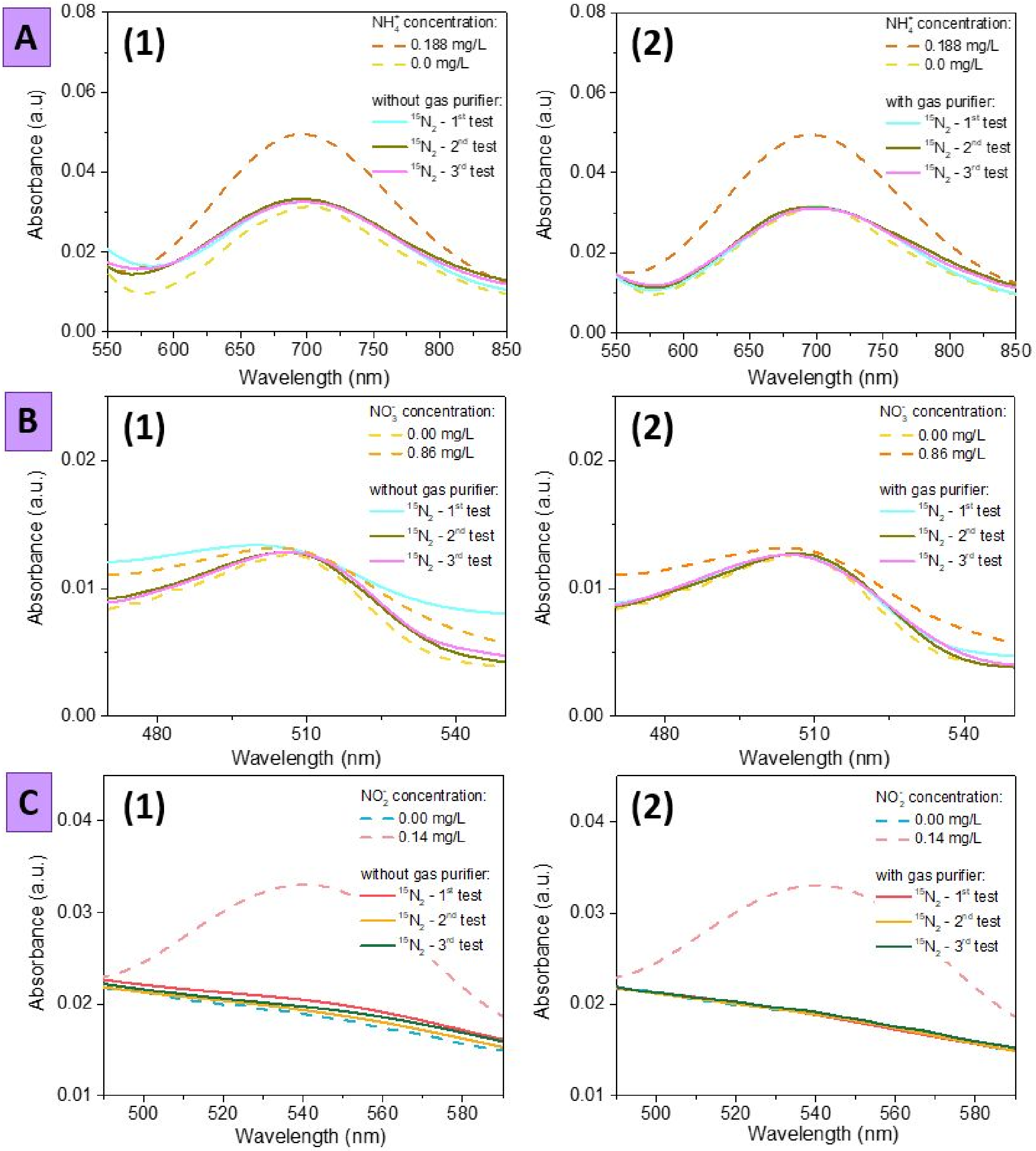


**Figure S19** Impacts of impurities removal/presence on nitrogen production: (A) – ammonia impurity case, (B) – nitrate impurity case, and (C) – nitrite impurity case. Results of repeated measurements (1) – without and (2) – with gas purifier.

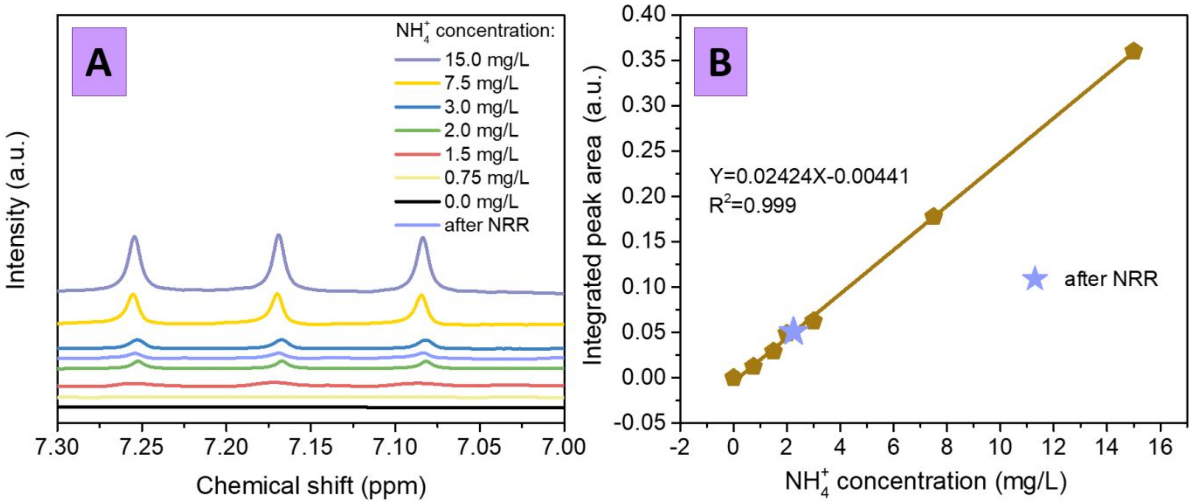


**Figure S20** $^1$H NMR spectra of standard ammonium solution ($^{14}NH_4Cl$) (A) and corresponding calibration curve (B), measured with utilization of gradually diluted $NH_4^+$ solution. The asterisk on the calibration curve (B) indicates the position of the "real" sample in which ammonia was obtained under the following conditions: Au grating/$TiB_2$@AuNPs photoelectrode, -0.2 V vs. RHE, 1 h, simulated sunlight illumination.

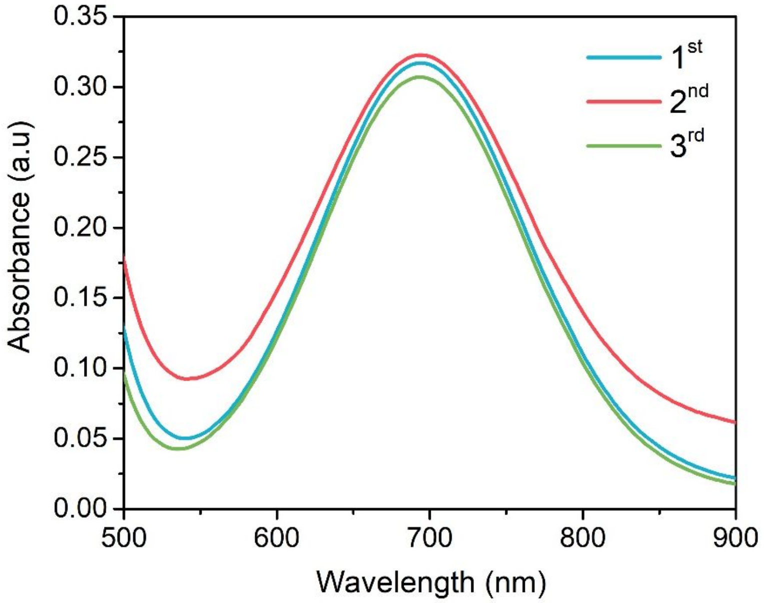


**Figure S21** UV-Vis absorption spectra of the photometric solution – revealing the $NH_3$ production under the utilization of Au grating/$TiB_2$@AuNPs photoelectrode after stability test (7 subsequent cycles of 3 h each) performed at -0.2 V (vs. RHE), under simulated sunlight illumination.

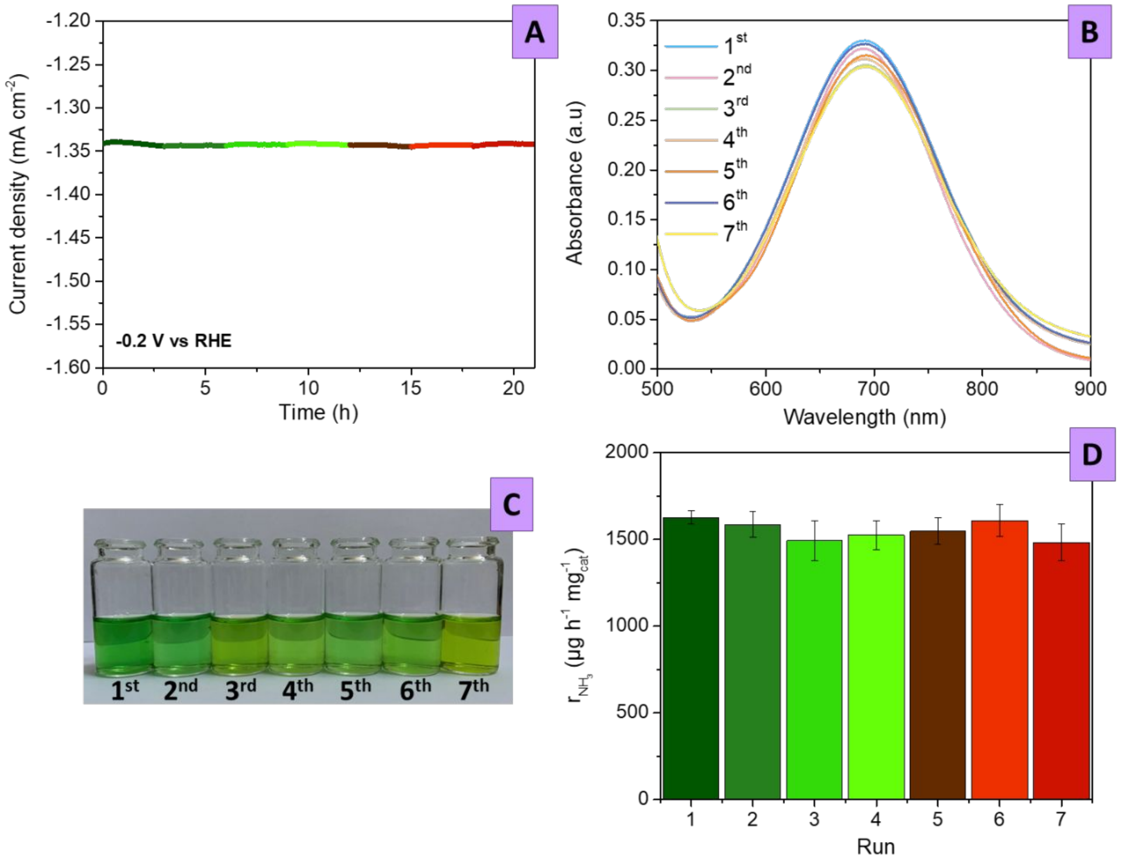


**Figure S22** Stability of Au grating/$TiB_2$@AuNPs (7 subsequent cycles of 3 h each) performed at -0.2 V (vs. RHE), under simulated sunlight illumination: (A) – chronoamperometry results; (B) – UV-Vis absorption spectra of the photometric solution – revealing the $NH_3$ production under the utilization of Au grating/$TiB_2$@AuNPs photoelectrode; (C) – corresponding photos of photometric solutions after its interaction with the reaction mixture; (D) – amounts of $NH_3$ produced after each cycle.

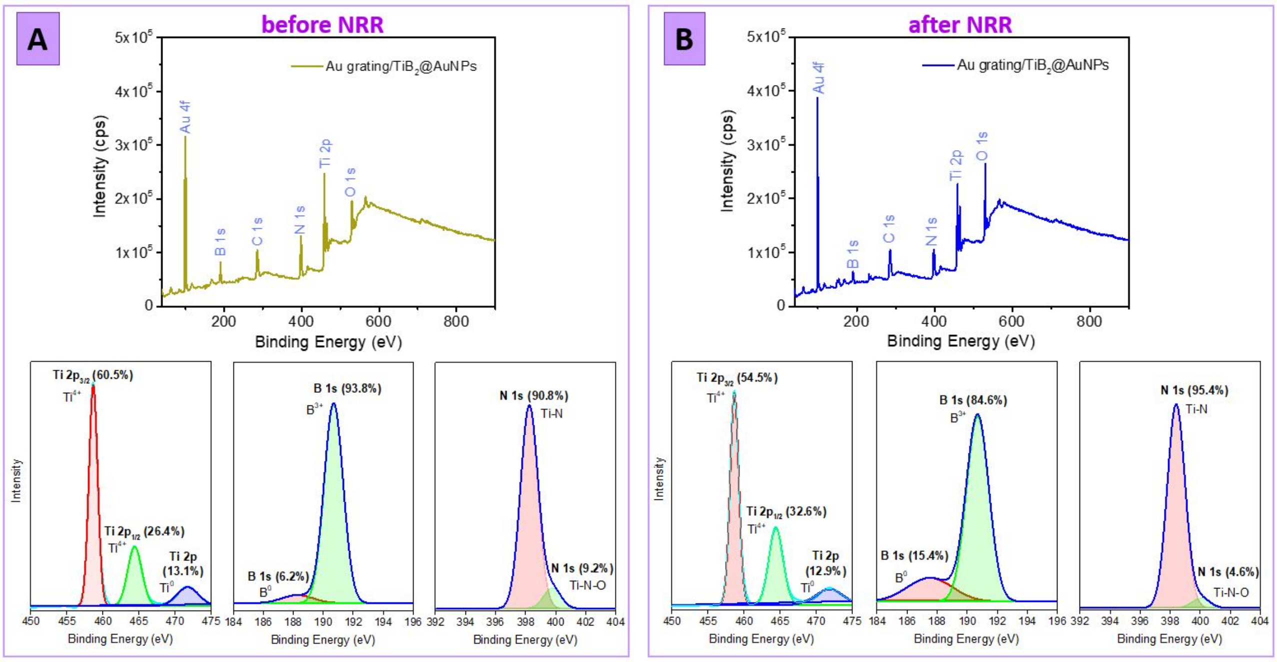


**C**

| | Element detection (at%) | | | | | |
|---|---|---|---|---|---|---|
| | Au 4f | B 1s | C 1s | N 1s | Ti 2p | O 1s |
| before NRR | 36.8 | 14.4 | 22.4 | 4.5 | 7.0 | 14.9 |
| after NRR | 38.9 | 11.3 | 23.2 | 4.0 | 5.1 | 17.5 |

**Figure S23** Survey XPS graphs and characteristic elements details measured on Au grating/$TiB_2$@AuNPs surface before (A) and after (B) samples utilization in NRR (H-type electrochemical cell, three-electrode system, sunlight illumination, applied potential -0.2V vs. RHE, $N_2$-saturated solution, duration - 21 h (7x3 h cycles)), (C) – elemental composition of samples surface calculated from survey XPS graphs.

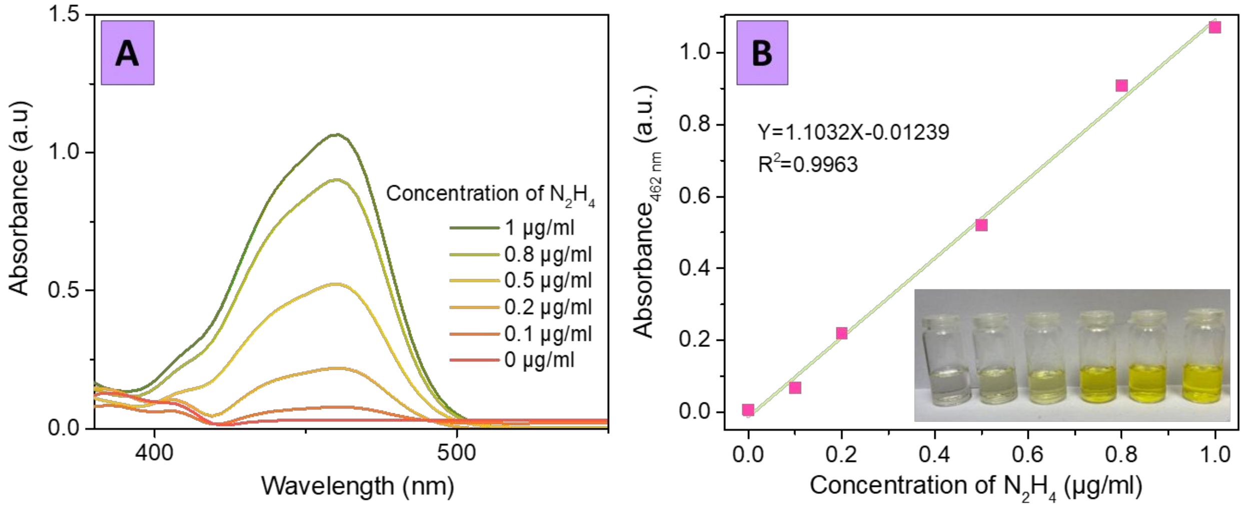


**Figure S24** (A) – UV-Vis absorption spectra of hydrazine photometric kit after the addition of various amounts of $N_2H_4$, (B) – calibration curve, prepared from UV-Vis absorption spectra and subsequently used to determine $N_2H_4$ production.

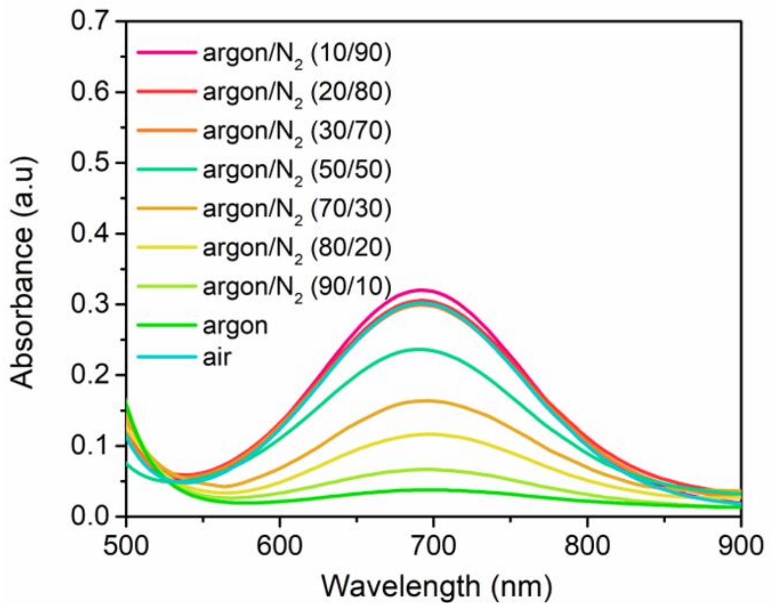


**Figure S25** UV-Vis spectra of ammonia photometric kit used for evaluation of NRR rate with the utilization of nitrogen/argon mixture or ambient air as a nitrogen source.

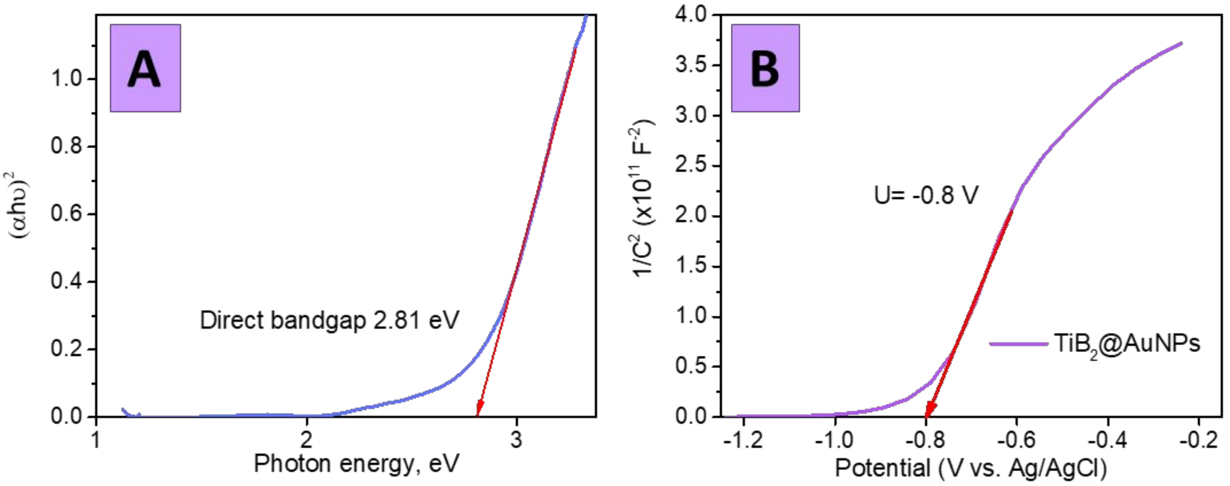


**Figure S26** Tauc plot (A) and Mott-Schottky plot (B) calculated from UV-Vis and EIS measurements of $TiB_2$@AuNPs flakes.

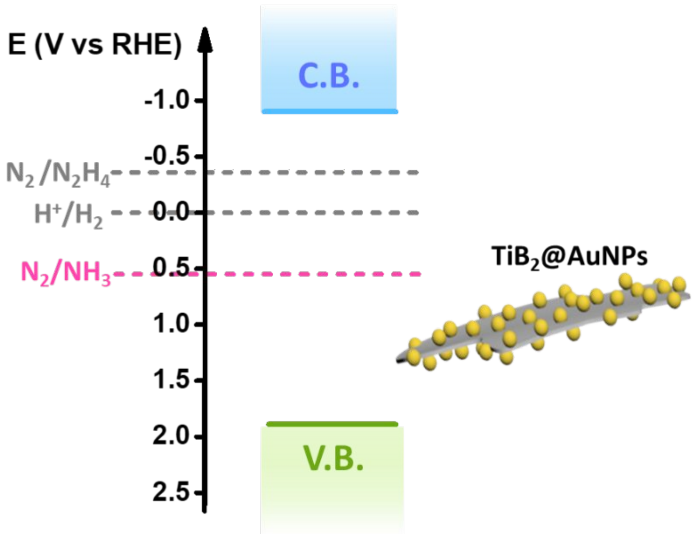


**Figure S27** Relationship between calculated bands structure of $TiB_2$@AuNPs flakes and potentials of NRR proceeding.

***Figs. S22 and S27 – description note***

The band gap of $TiB_2$@AuNPs nanostructures was calculated from the Tauc plot (Figure S26A), created from the UV-Vis spectrum (Figure S3 (A)). The band gap value was found to be . 2.81 eV. The type of $TiB_2$ semiconductor was determined in previous work [13]. The flat-band potentials of $TiB_2$@AuNPs ($E_{fb}$ vs. Ag/AgCl) were measured individually using the Schottky-Mott plot and found to be -0.8 V (Figure S26B). Using a potential difference of 0.21 V, the potential measured relative to the Ag/AgCl (3M KCl) reference was converted to the normal hydrogen electrode (NHE) potential. As a consequence, the predicted flat-band locations of $TiB_2$@AuNPs were

-0.6 V vs. NHE. Because the $E_{fb}$ for undoped n-type semiconductors is about 0.3 V below the conduction band minimum (CBM), the CBM of $TiB_2$@AuNPs was determined to be -0.9 V vs. NHE. The valence band maximum (VBM) was calculated using the location of the CBM and the band gap to be 1.91 V vs. NHE. Based on these results, Figure S27 presents the energy band values of $TiB_2$@AuNPs compared to $N_2/NH_3$, $H^+/H^0$, or $N_2/N_2H_4$ reaction potentials.

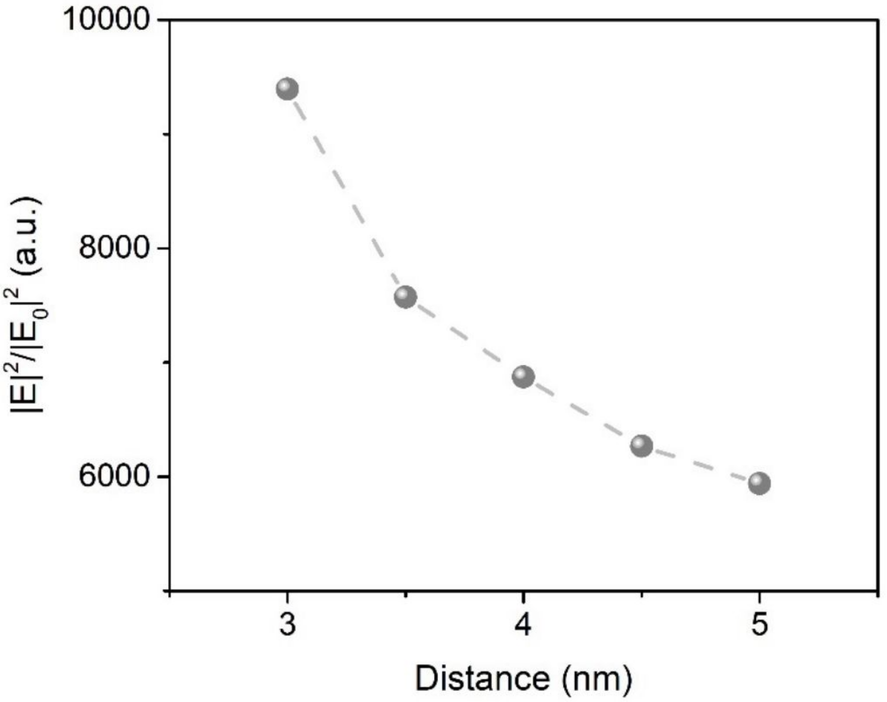


**Figure S28** Impact of spacer thickness of SPP-LSP coupling (local value of plasmon related electric field).

## References


(1) Zabelina, A.; Zabelin, D.; Miliutina, E.; Lancok, J.; Svorcik, V.; Chertopalov, S.; Lyutakov, O. Surface Plasmon-Polariton Triggering of $Ti_3C_2T_x$ MXene Catalytic Activity for Hydrogen Evolution Reaction Enhancement. *J. Mater. Chem. A* **2021**, *9* (33), 17770–17779. https://doi.org/10.1039/D1TA04505A.

(2) Hodgetts, R. Y. ; Kiryutin, A. S. ; Nichols, P. ; Du, H. L. ; Bakker, J. M. ; Macfarlane, D. R.; Simonov, A. N. Refining Universal Procedures for Ammonium Quantification via Rapid $^1$H NMR Analysis for Dinitrogen Reduction Studies. *ACS Energy Lett.* **2020**, 5 (3), 736–741. https://doi.org/10.1021/acsenergylett.9b02812.

(3) Huang, Y.; Ma, L.; Hou, M.; Li, J.; Xie, Z.; Zhang, Z. Hybridized Plasmon Modes and Near-Field Enhancement of Metallic Nanoparticle-Dimer on a Mirror. *Sci. Rep.* **2016**, *6* (1), 30011. https://doi.org/10.1038/srep30011.

(4) Johnson, R. Computational Chemistry Comparison and Benchmark Database, NIST Standard Reference Database 101, 2002. https://doi.org/10.18434/T47C7Z.

(5) Hutter, J.; Iannuzzi, M.; Schiffmann, F.; VandeVondele, J. Cp2k: Atomistic Simulations of Condensed Matter Systems. *WIREs Comp. Mol. Sci.* **2014**, *4* (1), 15–25. https://doi.org/10.1002/wcms.1159.

(6) Lippert, B.G.; Hutter, J.; Parrinello, M. A hybrid Gaussian and plane wave density functional scheme, *Mol. Phys*. **1997**, *92* (3), 477–488. https://doi.org/10.1080/002689797170220.

(7) VandeVondele, J.; Hutter, J. Gaussian Basis Sets for Accurate Calculations on Molecular Systems in Gas and Condensed Phases. *J. Chem. Phys.* **2007**, *127* (11), 114105. https://doi.org/10.1063/1.2770708.

(8) Goedecker, S.; Teter, M.; Hutter, J. Separable Dual-Space Gaussian Pseudopotentials. *Phys. Rev. B* **1996**, *54* (3), 1703–1710. https://doi.org/10.1103/PhysRevB.54.1703.

(9) Hartwigsen, C.; Goedecker, S.; Hutter, J. Relativistic Separable Dual-Space Gaussian Pseudopotentials from H to Rn. *Phys. Rev. B* **1998**, *58* (7), 3641–3662. https://doi.org/10.1103/PhysRevB.58.3641.

(10) Krack, M. Pseudopotentials for H to Kr Optimized for Gradient-Corrected Exchange-Correlation Functionals. *Theor. Chem. Acc.* **2005**, *114* (1), 145–152. https://doi.org/10.1007/s00214-005-0655-y.

(11) Perdew, J. P.; Burke, K.; Ernzerhof, M. Generalized Gradient Approximation Made Simple. *Phys. Rev. Lett.* **1996**, *77* (18), 3865–3868. https://doi.org/10.1103/PhysRevLett.77.3865.

(12) Caldeweyher, E.; Bannwarth, C.; Grimme, S. Extension of the D3 Dispersion Coefficient Model. *J. Chem. Phys.* **2017**, *147* (3), 034112. https://doi.org/10.1063/1.4993215.

(13) Zabelina, A.; Miliutina, E.; Zabelin, D.; Burtsev, V.; Buravets, V.; Elashnikov, R.; Neubertova, V.; Šťastný, M.; Popelková, D.; Lancok, J.; Chertopalov, S.; Paidar, M.; Trelin, A.; Michalcová, A.; Švorčík, V.; Lyutakov, O. Plasmon Coupling inside 2D-like $TiB_2$ Flakes for Water Splitting Half Reactions Enhancement in Acidic and Alkaline Conditions. *J. Chem. Eng.* **2023**, *454*, 140441. https://doi.org/10.1016/j.cej.2022.140441.
